# Ultraprocessing and presence of additives in commercially produced complementary foods in seven Southeast Asian countries: a cross-sectional study

**DOI:** 10.1016/j.ajcnut.2024.04.003

**Published:** 2024-05-28

**Authors:** Alissa M Pries, Eleonora Bassetti, Jane Badham, Philip Baker, Jessica Blankenship, Elizabeth K Dunford, Roland Kupka

**Affiliations:** 1 UNICEF East Asia Pacific Regional Office, Bangkok, Thailand; 2 JB Consultancy, Johannesburg, South Africa; 3 Faculty of Medicine and Health, Sydney School of Public Health, University of Sydney, Sydney, Australia; 4 The George Institute for Global Health, University of New South Wales, Sydney, New South Wales, Australia; 5 Department of Nutrition, Gillings Global School of Public Health, the University of North Carolina at Chapel Hill, Chapel Hill, United States

**Keywords:** complementary foods, ultraprocessed foods, additives, Southeast Asia, infant and young child nutrition

## Abstract

**Background:**

There has been a dramatic shift in food systems, and the consumption of commercially processed and packaged foods has grown globally, including among older infants and young children. Many of these products are ultraprocessed and contain additives, with concerning implications for the health and nutrition of children.

**Objectives:**

The study objectives were as follows: *1*) to assess the levels of processing among different commercially produced complementary food product (CPCF) categories marketed in the Southeast Asia region, *2*) to compare the nutrient content of CPCF products across levels of processing, and *3*) to assess the types of additives present in different CPCF categories.

**Methods:**

This cross-sectional study involved secondary analysis of a cross-sectional dataset of product label information from CPCF purchased in 2021 in Cambodia, Indonesia, Lao People’s Democratic Republic, Malaysia, Philippines, Thailand, and Viet Nam. Ingredient lists were reviewed to determine the level of processing—based on the Nova classification—and the presence of additives. Nutrient declaration panels were reviewed to determine total sugar, sodium, and total fat.

**Results:**

Nearly half of all CPCF were ultraprocessed, with total sugar and sodium content significantly higher among ultraprocessed CPCF than unprocessed/minimally processed products. Almost half of CPCF contained additives, with a median of 6 per product. More than 30% of all CPCF made use of cosmetic additives to enhance the products’ appearance, flavor, or texture, with emulsifiers, colors, and thickeners the most prevalent. Almost one-third of products contained additives not permitted in Codex Alimentarius standards and guidelines for CPCF.

**Conclusions:**

Findings from this study should alert national governments to both adopt and ensure enforcement of Codex guidance on additives and regulations enacted to encourage lower levels of processing for CPCF.

## Introduction

Recent decades have witnessed global growth in the production, marketing, and availability of commercially processed and packaged foods [1], leading to increased consumption of ultraprocessed foods (UPFs) [2,3]. The diets of older infants and young children are not isolated from this transition—the global market for commercially processed and packaged foods marketed for consumption by this age group has grown from ∼10 billion USD to 18 billion USD between 2010 and 2022 [4] and UPF make up a substantial proportion of infant and young child (IYC) diets in many contexts [5,6].

High consumption of UPF has been flagged as a public health concern because of associated negative impacts on health and nutrition outcomes [7,8]. UPF are often high in total sugar, sodium, and unhealthy fats [6,9,10] and can lead to excessive intake of these nutrients. Preschoolers residing in the United States who were the highest consumers of UPF were 3 times more likely to exceed global recommendations for added sugar intake than lower consumers [6]. High consumption of UPF can also displace consumption of other micronutrient-rich foods—the same study of United States preschoolers noted an inverse relationship between UPF consumption and consumption of unprocessed/minimally processed fruits and milk [6], and a study among 4–5 y olds in Spain found a negative association between intakes of 15 of 20 micronutrients assessed and UPF consumption [11].

A defining feature of UPF, based on Nova classification, is the presence of cosmetic additives, such as flavor enhancers, colors, and emulsifiers [12]. The linkages between UPF consumption and negative impacts on gut microbiome and inflammatory disease [13,14] have been increasingly researched, with evidence indicating a potential pathway via certain additives common in UPF [15,16]. The first years of life are a critical period for the maturation of IYC microbiomes; dietary patterns that may have adverse effects on microbiome development, such as high consumption of UPF, and that could potentially inhibit child growth and development [17] are under growing scrutiny.

There has been a call to better understand the levels of processing and presence of additives in commercial products marketed for older IYC feeding [18]. Although prior research has assessed the degree of processing among commercially produced complementary food products (CPCFs) available in the European market [19,20], there is a paucity of information from markets in other geographic regions, particularly in low- and middle-income countries and almost no research types of additives used in CPCF.

This study aimed to build an understanding of the characteristics of CPCF marketed in the Southeast Asia region. The objectives of this study were as follows: *1*) to assess the levels of processing among different CPCF product categories, *2*) to compare the nutrient content of CPCF across levels of processing, *3*) to assess the types of additives present in different CPCF categories.

## Methods

This study involved secondary analysis of a cross-sectional dataset of product label information from CPCF available in 7 countries across the Southeast Asia region. The primary study from which the data set was sourced was conducted by the UNICEF East Asia Pacific Region, and the countries included national offices where national researchers could be engaged. Products were identified and purchased in 2021 across Cambodia (Phnom Penh), Indonesia (Jakarta), Lao People’s Democratic Republic (PDR) (Vientiane), Malaysia (Kuala Lumpur), Philippines (Manila), Thailand (Bangkok), and Viet Nam (Hanoi).

### Product sampling

Detailed explanation of the methodology used to identify and sample CPCF has been previously published elsewhere [[21], [22], [23]], and methods are summarized here. To identify products sold in Manila, a product list from a 2020 study [24] was obtained, and all products included in this list were purchased from online retailers. If a product was no longer available, it was excluded from the study. Identification of CPCF products in the remaining cities involved 2 steps. In the first step, retailers selling CPCF in each city were scoped and sampled. Scoping of retailers involved communications with local experts in IYC feeding in each city to identify the types and names of retailers that are present and were likely to sell CPCF (i.e., chain supermarkets, pharmacies, independent groceries, convenience stores, infant stores), as well as a web-based search. From this list, all independent stores and one physical store of any chain retailer were purposively sampled. In addition, online stores for any of the sampled retailers were also included. In the second step, the physical stores and online stores were visited, CPCF were identified, and all products meeting the definition of CPCF were purchased.

In all 7 cities, CPCF were defined as any food/beverage product that was marketed as suitable for feeding an older IYC <3 y of age by meeting any of the 4 following criteria: *1*) were recommended for introduction at the age of <3 y; *2*) were labeled with the words ‘baby,’ ‘infant,’ ‘toddler,’ ‘young child’ or a synonym; *3*) had a label with an image of a child who appeared to be <3 y of age or who was feeding with a bottle; or *4*) were in any other way presented as being suitable for children aged <3 y [25]. Nutritional supplements and commercial milk formula (i.e., infant formula, follow-on formula, and growing-up/toddler kinds of milk) were not included. Products whose label information was not presented in either the local language of the country of purchase or in English were excluded. Once products were purchased, duplicates of products at the city level were removed, photographs were taken of each product, and the label information was extracted and entered into a database.

### Data management and analysis

Based on an adaption categorization from the WHO [26], CPCF were categorized into 15 product subcategories across the following 5 main categories: *1*) dry/instant cereals; *2*) ready-to-eat purees/meals; *3*) finger foods/snacks; *4*) beverages; and *5*) condiments.

The ingredient lists of all CPCF were reviewed to identify the presence of additives. Additives were considered as any ingredient defined as an additive by the Codex Alimentarius (Codex) General Standard for Food Additives, specifically: ‘Any substance not normally consumed as a food by itself and not normally used as a typical ingredient of the food, whether or not it has nutritive value, the intentional addition of which to food for a technological (including organoleptic) purpose in the manufacture, processing, preparation, treatment, packing, packaging, transport or holding of such food results, or may be reasonably expected to result (directly or indirectly), in it or its by-products becoming a component of or otherwise affecting the characteristics of such foods.’ [27]. Ingredients identified as additives were categorized into 27 Codex functional classes as follows: acidity regulators, anticaking agents, antifoaming agents, antioxidants, bleaching agents, bulking agents, carbonating agents, carriers, colors, color retention agents, emulsifiers, emulsifying salts, firming agents, flavor enhancers, flour treatment agents, foaming agents, gelling agents, glazing agents, humectants, packaging gases, preservations, propellants, raising agents, sequestrants, stabilizers, sweeteners, and thickeners [28]. Some additives carry more than 1 function in Codex classification. It is impossible to determine the function intended by the manufacturer based on label information; however, the presence of an additive in a product would provide all of these functions. Therefore, for this analysis, additives were categorized by all associated functions. Due to the potential negative effect on human health, the presence of 2 additional types of additives was assessed—phosphate additives [29] and nitrates [30,31]. Phosphate additives were defined as inorganic phosphates following methods previously used for CPCF products in the United States [32]. A list of the search terms used to identify each functional class of additives, as well as phosphate additives and nitrates, across CPCF ingredient lists is provided in Supplemental Table 1.

Codex was established by the WHO and the FAO to develop international food standards, guidelines, and codes of practice to protect consumer health and ensure fair trade for the food industry. The standards and guidelines established by Codex often serve as the basis for national level food standards. Four CPCF-specific documents were reviewed to identify additives permitted in CPCF by Codex, these included the following: *1*) Advisory Lists of Nutrient Compounds for Use in Foods for Special Dietary Uses intended for Infants and Children (CAC/GL 10-1979) [33], *2*) Standard for processed cereal-based foods for infants and young children (Codex Stan 74-1981) [34], *3*) Standard for canned infant foods (Codex Stan 73-1981) [35], and *4*) Guidelines on Formulated Complementary Foods for older infants and young children (CAC/GL 8-1991) [36]. Permitted additives in Codex Stan 74-1981 were considered permitted for products within the dry/instant cereals main category and cereal-based finger foods/snacks product subcategory. Permitted additives in Codex Stan 73-1981 were considered permitted for products within the purees/meals main category. As CAC/GL 8-1991 states, ‘Food additives and flavorings listed in the Standard for Processed Cereal-Based Foods for Infants and Young Children (CODEX STAN 74-1981) and the Standard for Canned Baby Foods (CODEX STAN 73-1981) may be used in Formulated Complementary Foods to the maximum limits given in those standards’ [36], the permitted additives listed in these 2 standards were considered permitted in all remaining CPCF categories. Finally, the additives noted as permitted in CAC/GL 10-1979 were considered as permitted additives in all CPCF categories. The specific additives permitted by Codex are detailed in Supplemental Table 2. Ingredient lists from the CPCF labels were reviewed to identify products containing these permitted additives, and the proportion of products that contained additives beyond those permitted was calculated.

Using the Nova classification system, ingredient lists of all CPCF were assessed to categorize products across 4 groups by level of processing [12,37]. Unprocessed/minimally processed foods (group 1) were defined as products that only had unprocessed foods listed as ingredients. As the Nova classification system states that unprocessed/minimally processed foods may include ‘foods with vitamins and minerals added,’ unprocessed/minimally processed CPCF that listed the addition of fortificants in their ingredient lists were categorized as unprocessed/minimally processed [37]. Processed culinary ingredients (group 2) were defined as products containing only ingredients derived from unprocessed/minimally processed foods by industrial processes, including pressing, centrifuging, refining, extracting, or mining. These include oils, salts, and sugars and may contain additives to prolong duration, protect original properties, or prevent proliferation of microorganisms and added vitamins or minerals. Processed foods (group 3) were defined as products that included unprocessed foods but also processed culinary ingredients (sugars, salts, fats, or oils) in their ingredient lists. These foods may contain additives that prolong product duration, protect original properties, or prevent proliferation of microorganisms. Ultraprocessed foods (group 4) were defined as products that included ingredient markers, specifically: inverted sugar, dextrose, fructose, lactose, glucose, maltodextrin, concentrated juice, syrup, protein concentrate, protein isolate, whey protein, soy protein, wheat gluten, casein, fiber, maltitol, sorbitol, interesterified, hydrogenated or fractionated oil/fat, gelatin, pectin, gums, mechanically separated meat, milk whey, dairy product solids, modified starch, monosodium glutamate, artificial essence/flavor, sucralose, aspartame, acesulfame, cyclamate, saccharin, Stevia [37]. Additionally, to ensure that all possible markers of UPF were identified in accordance with the Nova classification definition [38], CPCF that listed any ‘cosmetic’ additives, defined as additives that function to enhance flavor, color, or texture, in their ingredient list were categorized as ultraprocessed. Cosmetic additives included the following functional classes of additives: antifoaming agents, bulking agents, carbonating agents, colors, emulsifiers, emulsifying salts, flavor enhancers, foaming agents, gelling agents, glazing agents, sweeteners, and thickeners [12].

The quantity of sodium (mg), total sugar (g), and total fat (g) per 100 kcal of each CPCF was calculated based on declared nutrient content. Products whose labels did not declare sodium, total sugar, or total fat content were excluded from the analysis of that specific nutrient.

Analysis was conducted using Stata (version 18.0). Descriptive statistics were calculated and summarized using proportions and frequencies. Nutrient content was presented as medians and IQRs. Kruskal-Wallis tests, with Dunn’s post hoc tests, were used to test differences in medians between groups. Significance was set at *P* value of <0.05.

## Results

A total of 1635 CPCF available across the 7 cities were included in this study: Bangkok *n* = 206, Hanoi n=242, Jakarta *n* = 272, Kuala Lumpur *n* = 388, Manila *n* = 182, Phnom Penh *n* = 227, and Vientiane *n* = 118 (Table 1, Supplemental Figure 1). The most common overall CPCF category was finger foods and snacks, making up >1/3 (37.1%) of all CPCF, with cereal-based snacks being the most common subcategory, with confectionery snacks and fruit snacks being less common. Dry or instant cereal and ready-to-eat purees/meals each made up just >1/4 of products (29.6% and 28.1%, respectively), with fruit purees being the most common. Although condiments were not commonly identified, 3.2% of CPCF were seasoning powders or flosses specifically marketed as suitable for children aged <3 y.

**TABLE 1:** Commercially produced complementary food product categories (*N* = 1635)

Category	% (n)
1. Dry/instant cereals	29.6 (484)
2. Ready-to-eat purees/meals	28.1 (460)
2.1 Dairy-based desserts	2.6 (42)
2.2 Fruit purées	15.1 (246)
2.3 Vegetable only purées	1.5 (25)
2.4 Vegetable and cereals purées	1.4 (22)
2.5 Purées with cheese, meat or fish	5.5 (90)
2.6 Meat/fish/cheese-based meals	2.1 (34)
2.7 Vegetable-based meals	0.1 (1)
3. Finger foods/snacks	37.1 (606)
3.1 Confectionery finger foods/snacks	3.1 (50)
3.2 Fruit finger foods/snacks	0.5 (8)
3.3 Cereal-based finger foods/snacks	33.5 (548)
4. Beverages	1.0 (17)
4.1 Fruit/vegetable juices	0.9 (15)
4.2 Milk/milk alternatives with added sugar/sweetener	0.1 (2)
5. Condiments	4.2 (68)
5.1 Oils	0.9 (15)
5.2 Seasonings, powders, flosses	3.2 (53)

**FIGURE 1:**
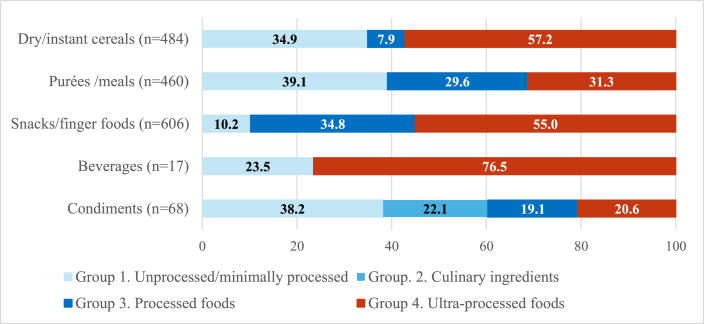
Level of processing among commercially produced complementary foods by product category

Almost half (47.7%, *n* = 781) of all CPCF were ultraprocessed, whereas 24.3% (*n* = 398) were processed, 0.9% (*n* = 15) were processed culinary ingredients, and 27.0% (*n* = 441) were unprocessed/minimally processed. Level of processing varied substantially by product category (Figure 1). Over 3/4 (76.5%) of beverages and >1/2 of snacks/finger foods and dry-instant cereals were ultraprocessed (55.0% and 57.2%, respectively). However, >1/3 of dry/instant cereals, condiments, and purées/meals (34.9%, 38.2%, and 39.1%, respectively) were unprocessed/minimally processed, indicating a wide variance in the level of processing between product categories. Variation in level of processing was also noted within product categories. Among snacks/finger foods, >3/4 (82.0%, *n* = 41) of confectionery snacks and >1/2 half (53.3%, *n* = 292) of cereal-based snacks were ultraprocessed, whereas no fruit snacks were ultraprocessed. Although >1/3 of purées/meals were ultraprocessed (31.3%, *n* = 144), >1/2 of products in the dairy-based desserts subcategory (52.4%, *n* = 22) within purées/meals were ultraprocessed. More than 3/4 (84%, *n* = 21) of vegetable only purees and almost 2/3 (63.6%, *n* = 14) of vegetable and cereals purees were unprocessed/minimally processed. The proportion of CPCF that were ultraprocessed also varied by country (Supplemental Table 3)—whereas only 18.9% of CPCF were ultraprocessed in Thailand, ∼2/3 of products were ultraprocessed in the Philippines and Viet Nam (60.4% and 63.2%, respectively).

Total sugar, sodium, and total fat contents per 100 kcal across product categories and by level of processing are presented in Table 2. Among dry/instant cereals, purees/meals, and snacks/finger foods, ultraprocessed products contained a significantly greater median amount of total sugar per 100 kcal of product compared with both processed and unprocessed/minimally processed products [3.3g/100 kcal compared with 0.8 (*P* < 0.001) and 0.6 (*P* < 0.001) g/100 kcal, respectively, for dry/instant cereals; 14.3 g/100 kcal compared with 9.9 (*P* = 0.043) and 10.0 g/100 kcal (*P* = 0.030), respectively, for purees/meals; and 4.0 g/100 kcal compared with 2.2 (*P* < 0.001) and 0.0 (*P* < 0.001) g/100 kcal, respectively for snacks/finger foods]. Sodium per 100 kcal was significantly greater among ultraprocessed dry/instant cereals and snacks/finger foods compared with unprocessed/minimally processed products (36 mg/100 kcal compared with 2 mg/100 kcal; *P* < 0.001) for dry/instant cereals; 20 mg/100 kcal compared with 0 mg/100kcal (*P* < 0.001) for snacks/finger foods) but similar to processed products for dry/instant cereals (36 mg/100 kcal compared with 24 mg/100 kcal; *P* = 0.833) and processed snacks/finger foods had slightly higher sodium contents compared to ultraprocessed products (33 mg/100 kcal compared with 20 mg/100 kcal (*P* = 0.042). Among condiments, total fat per 100 kcal was highest among processed culinary ingredients, as these products were oils.

**TABLE 2:** Median total sugar, sodium, and total fat contents of commercially produced complementary foods across levels of processing by product category^1^

	Total sugar (g/100 kcal)	Sodium (mg/100 kcal)	Total fat (g/100 kcal)
Dry/instant cereals^2^			
Group 1. Unprocessed/minimally processed	0.6 [0–1.2]^a^	2 [0–6]^a^	0.5 [0.3–0.7]^a^
Group 2. Processed culinary ingredients	—	—	—
Group 3. Processed foods	0.8 [0.2–2.7]^a^	24 [8–70]^b^	0.6 [0.4–1.6]^a^
Group 4. Ultraprocessed foods	3.3 [2.0–6.2]^b^	36 [[14], [15], [16], [17], [18], [19], [20], [21], [22], [23], [24], [25], [26], [27], [28], [29], [30], [31], [32], [33], [34], [35], [36], [37], [38], [39], [40], [41], [42], [43], [44], [45], [46], [47], [48], [49], [50], [51], [52], [53], [54], [55], [56], [57], [58], [59], [60]]^b^	1.9 [1.3–2.4]^b^
Purees/meals^3^			
Group 1. Unprocessed/minimally processed	10.0 [4.3–17.2]^a^	25 [[7], [8], [9], [10], [11], [12], [13], [14], [15], [16], [17], [18], [19], [20], [21], [22], [23], [24], [25], [26], [27], [28], [29], [30], [31], [32], [33], [34], [35], [36], [37], [38], [39], [40], [41], [42], [43], [44], [45], [46], [47], [48], [49], [50]]^a^	0.4 [0.1–1.4]^a^
Group 2. Processed culinary ingredients	—	—	—
Group 3. Processed foods	9.9 [3.2–17.8]^a^	22 [[8], [9], [10], [11], [12], [13], [14], [15], [16], [17], [18], [19], [20], [21], [22], [23], [24], [25], [26], [27], [28], [29], [30], [31], [32], [33], [34], [35], [36], [37], [38], [39], [40], [41], [42], [43], [44], [45], [46], [47], [48], [49]]^a^	2.0 [0.5–3.6]^b^
Group 4. Ultraprocessed foods	14.3 [9.2–16.7]^b^	19 [[6], [7], [8], [9], [10], [11], [12], [13], [14], [15], [16], [17], [18], [19], [20], [21], [22], [23], [24], [25], [26], [27], [28], [29], [30], [31], [32], [33]]^b^	0.9 [0.0–2.2]^a^
Snacks/finger foods^4^			
Group 1. Unprocessed/minimally processed	0.0 [0.0–2.3]^a^	0 [0–6]^a^	0.0 [0.0–0.3]^a^
Group 2. Processed culinary ingredients	—	—	—
Group 3. Processed foods	2.2 [0.6–3.3]^a^	33 [3–71]^b^	0.5 [0.0–2.8]^b^
Group 4. Ultraprocessed foods	4.0 [2.8–5.7]^b^	20 [[2], [3], [4], [5], [6], [7], [8], [9], [10], [11], [12], [13], [14], [15], [16], [17], [18], [19], [20], [21], [22], [23], [24], [25], [26], [27], [28], [29], [30], [31], [32], [33], [34], [35], [36], [37], [38], [39], [40], [41], [42], [43], [44], [45], [46], [47], [48], [49], [50]]^c^	1.7 [0.0–2.9]^b^
Beverages^5^			
Group 1. Unprocessed/minimally processed	17.9 [17.3–20.4]^a^	43 [[18], [19], [20], [21], [22], [23], [24], [25], [26], [27], [28], [29], [30], [31], [32], [33], [34], [35], [36], [37], [38], [39], [40], [41], [42], [43], [44], [45], [46], [47], [48], [49], [50], [51], [52], [53], [54], [55], [56], [57]]^a^	0.1 [0.1–0.2]^a^
Group 2. Processed culinary ingredients	—	—	—
Group 3. Processed foods	—	—	—
Group 4. Ultraprocessed foods	23.2 [21.6–23.2]^b^	0 [0–13]^b^	0.0 [0.0–0.0]^a^
Condiments^6^			
Group 1. Unprocessed/minimally processed	0.0 [0.0–0.0]^a^	45 [17–208]^a^	0.4 [0.0–4.0]^a^
Group 2. Processed culinary ingredients	0.0 [0.0–0.0]^a^	0 [0–0]^b^	11.1 [11.1–11.1]^b^
Group 3. Processed foods	6.0 [4.0–6.8]^b^	196 [135–800]^a^	2.9 [2.0–3.6]^a^
Group 4. Ultraprocessed foods	11.1 [11.1–11.1]^b^	200 [156–1250]^a^	1.1 [0.6–2.2]^a^

1 Values presented as median [IQR]; comparisons of medians were made using Kruskal-Wallis and Dunn’s post hoc tests - labeled medians within product categories in a column without a common letter differ, *p* < 0.05.

2 Dry/instant cereal products containing nutrient content information on labels: sugar – group 1 (*n* = 117), group 3 (*n* = 28), group 4 (*n* = 194); sodium – group 1 (*n* = 149), group 3 (*n* = 36), group 4 (275); total fat – group 1 (*n* = 168), group 3 (*n* = 38), group 4 (*n* = 277).

3 Puree products containing nutrient content information on labels: sugar – group 1 (*n* = 156), group 3 (*n* = 120), group 4 (*n* = 123); sodium – group 1 (*n* = 175), group 3 (*n* = 130), group 4 (*n* = 128); total fat – group 1 (*n* = 180), group 3 (*n* = 136), group 4 (*n* = 134).

4 Snack/finger food products containing nutrient content information on labels: group 1 (*n* = 60), group 3 (*n* = 199), group 4 (*n* = 289); sodium – group 1 (*n* = 60), group 3 (*n* = 206), group 4 (*n* = 324); total fat – group 1 (*n* = 62), group 3 (*n* = 211), group 4 (*n* = 333).

5 Beverage products containing nutrient content information on labels: group 1 (*n* = 4), group 3 (*n* = 0), group 4 (*n* = 9); sodium – group 1 (*n* = 4), group 3 (*n* = 0), group 4 (*n* = 9); total fat – group 1 (*n* = 4), group 3 (*n* = 0), group 4 (*n* = 11).

6 Condiment products containing nutrient content information on labels: group 1 (*n* = 18), group 2 (*n* = 5), group 3 (*n* = 10), group 4 (*n* = 3); sodium – group 1 (*n* = 22), group 2 (*n* = 7), group 3 (*n* = 12), group 4 (*n* = 7); total fat – group 1 (*n* = 23), group 2 (*n* = 11), group 3 (*n* = 12), group 4 (*n* = 7).

Almost half (47.9%, *n* = 783) of all products contained additives, and 1/3 (34.8%, *n* = 569) specifically contained cosmetic additives. However, the presence of additives varied substantially across CPCF categories (Table 3). Nearly 1/2 (49.8%) of all CPCF dry/instant cereals and snacks/finger foods (45.1%) and ∼1/4 (23.5%) of beverages contained cosmetic additives, compared with 9.4% of purees/meals and 11.8% of condiments. The proportion of CPCF with cosmetic additives also varied by country, ranging from 16.5% of products in Thailand to ∼1/2 of products in Cambodia (40.5%), Viet Nam (43.8%), and Indonesia (45.6%) (Supplemental Table 4). Emulsifiers, colors, and thickeners were the most common cosmetic additives, and acidity regulators, anticaking agents, antioxidants, firming agents, flour treatment agents, sequestrants, and stabilizers were the most common noncosmetic additives. Among CPCF that contained a cosmetic additive, products contained a median of 1 cosmetic additive, with a maximum of 19 cosmetic additives. Among CPCF containing any additives, products contained a median of 6 additives, with a maximum of 43 additives present. No CPCF were identified to contain nitrates, and 7.1% (*n* = 115) were found to contain a phosphate additive.

**TABLE 3:** Presence of additives in commercially produced complementary foods by product category^1^

	Dry/instant cereals *n* = 484 (%)	Purées/meals *n* = 460 (%)	Snacks/finger foods *n* = 606 (%)	Beverages *n* = 17 (%)	Condiments n = 68 (%)	All products *n* = 1635 (%)
Cosmetic additives						
Antifoaming agent	2.3 (11)	1.3 (6)	1.0 (0)	11.8 (2)	0.0 (0)	1.2 (19)
Bulking agent	0.2 (1)	0.2 (1)	0.5 (3)	23.5 (4)	0.0 (0)	0.6 (9)
Carbonating agent	1.0 (0)	0.0 (0)	0.0 (0)	1.0 (0)	0.0 (0)	0.0 (0)
Color	29.8 (144)	1.7 (8)	24.8 (150)	5.9 (1)	0.0 (0)	18.6 (304)
Emulsifier	29.8 (144)	8.0 (37)	22.1 (134)	23.5 (4)	0.0 (0)	19.5 (319)
Emulsifying salt	9.7 (47)	0.9 (4)	3.0 (18)	1.0 (0)	0.0 (0)	4.2 (69)
Flavor enhancer	4.1 (20)	0.4 (2)	2.6 (16)	11.8 (2)	0.0 (0)	2.5 (40)
Foaming agent	1.0 (0)	0.7 (3)	0.7 (4)	0.0 (0)	0.0 (0)	0.4 (7)
Gelling agent	1.0 (0)	0.9 (4)	0.5 (3)	23.5 (4)	0.0 (0)	0.7 (11)
Glazing agent	2.3 (11)	1.5 (7)	0.5 (3)	23.5 (4)	0.0 (0)	1.5 (25)
Sweetener	1.0 (0)	0.0 (0)	0.0 (0)	11.8 (2)	0.0 (0)	0.1 (2)
Thickener	19.4 (94)	4.8 (22)	14.7 (89)	23.5 (2)	11.8 (8)	13.3 (217)
Other additives						
Acidity regulator	35.3 (171)	15.9 (73)	36.8 (223)	41.2 (7)	1.5 (1)	29.1 (475)
Anticaking agent	30.4 (147)	0.2 (1)	24.9 (151)	1.0 (0)	0.0 (0)	18.3 (299)
Antioxidant	39.1 (189)	29.4 (135)	31.4 (190)	41.2 (7)	1.5 (1)	31.9 (522)
Bleaching agent	1.0 (0)	1.0 (0)	1.0 (0)	1.0 (0)	1.5 (1)	0.1 (1)
Carrier	4.6 (22)	0.2 (1)	0.8 (5)	23.5 (4)	0.0 (0)	2.0 (32)
Color retention agent	7.0 (34)	14.1 (65)	6.6 (40)	41.2 (7)	1.5 (1)	9.0 (147)
Firming agent	31.2 (151)	0.4 (2)	17.7 (107)	11.8 (2)	0.0 (0)	16.0 (262)
Flour treatment agent	50.4 (244)	23.5 (108)	31.9 (193)	11.8 (2)	1.5 (1)	33.5 (548)
Humectant	10.3 (50)	2.4 (11)	3.0 (18)	23.5 (4)	0.0 (0)	5.1 (83)
Packaging gas	1.0 (0)	0.0 (0)	0.3 (2)	0.0 (0)	0.0 (0)	0.1 (2)
Preservative	4.1 (20)	0.4 (2)	4.3 (26)	0.0 (0)	1.5 (1)	3.0 (49)
Propellant	1.0 (0)	0.0 (0)	0.3 (2)	0.0 (0)	0.0 (0)	0.1 (2)
Raising agent	2.5 (12)	0.2 (1)	13.9 (84)	0.0 (0)	0.0 (0)	5.9 (97)
Sequestrant	17.6 (85)	28.0 (129)	8.6 (52)	41.2 (7)	1.5 (1)	16.8 (274)
Stabilizer	31.8 (154)	4.6 (21)	26.2 (159)	23.5 (4)	0.0 (0)	20.7 (338)
Any cosmetic additive	49.8 (241)	9.4 (43)	45.1 (273)	23.5 (4)	11.8 (8)	34.8 (569)
Any additive	58.3 (282)	34.1 (157)	54.0 (327)	52.9 (9)	11.8 (8)	47.9 (783)

1 Values presented in %(*n*)

The proportion of CPCF containing additives not permitted by Codex is detailed in Table 4. Overall, ∼1/3 (30.2%, *n* = 494) contained at least one nonpermitted additive. The most common functional classes of nonpermitted additives in CPCF included emulsifiers (14.9%, *n* = 243), antioxidants (13.8%, *n* = 225), thickeners (12.0%, *n* = 196), and acidity regulators (11.8%, *n* = 193). Dry/instant cereals (42.8%, *n* = 207) and snacks/finger foods (37.5%, *n* = 227) were the most common CPCF categories to contain nonpermitted additives. Almost ¼ quarter of beverages (23.5%) contained nonpermitted additives, with the most common functional classes of nonpermitted additives differing from other CPCF product categories—primarily bulking agents, carriers, gelling agents, glazing agents, humectants, stabilizers, and thickeners. The proportion of CPCF containing nonpermitted additives varied by country—18.5% of products in Thailand contained nonpermitted additives compared with 53.7% of products in Indonesia (Supplemental Table 5).

**TABLE 4:** Proportion of commercially produced complementary foods containing nonpermitted additives by product category^1^

	Dry/instant cereals *n* = 484 (%)	Purées/meals *n* = 460 (%)	Snacks/finger foods *n* = 606 (%)	Beverages *n* = 17 (%)	Condiments *n* = 68 (%)	All products *n* = 1635 (%)
Acidity regulator	15.7 (76)	7.4 (34)	13.4 (81)	11.8 (2)	0.0 (0)	11.8 (193)
Anticaking agent	5.8 (28)	0.2 (1)	1.5 (9)	0.0 (0)	0.0 (0)	2.3 (38)
Antifoaming agent	0.0 (0)	0.0 (0)	0.0 (0)	0.0 (0)	0.0 (0)	0.0 (0)
Antioxidant	25.6 (124)	3.9 (18)	13.5 (82)	0.0 (0)	1.5 (1)	13.8 (225)
Bleaching agent	0.0 (0)	0.0 (0)	0.0 (0)	0.0 (0)	1.5 (1)	0.1 (1)
Bulking agent	0.0 (0)	0.2 (1)	0.5 (3)	23.5 (4)	0.0 (0)	0.5 (8)
Carbonating agent	0.0 (0)	0.0 (0)	0.0 (0)	0.0 (0)	0.0 (0)	0.0 (0)
Carrier	4.1 (20)	0.2 (1)	0.8 (5)	23.5 (4)	0.0 (0)	1.8 (30)
Color	3.7 (18)	1.7 (8)	8.8 (53)	5.9 (1)	1.5 (1)	5.0 (81)
Color retention agent	3.1 (15)	0.0 (0)	0.2 (1)	0.0 (0)	0.0 (0)	1.0 (16)
Emulsifier	25.8 (125)	6.5 (30)	13.9 (84)	23.5 (4)	0.0 (0)	14.9 (243)
Emulsifying salt	9.7 (47)	0.9 (4)	1.3 (8)	0.0 (0)	0.0 (0)	3.6 (59)
Firming agent	4.1 (20)	0.4 (2)	2.6 (14)	11.8 (2)	0.0 (0)	2.5 (40)
Flavor enhancer	4.1 (20)	0.4 (2)	2.6 (14)	11.8 (2)	0.0 (0)	2.5 (40)
Flour treatment agent	5.2 (25)	0.2 (1)	0.7 (4)	0.0 (0)	1.5 (1)	1.9 (31)
Foaming agent	0.0 (0)	0.7 (3)	0.7 (4)	0.0 (0)	0.0 (0)	0.4 (7)
Gelling agent	0.0 (0)	0.2 (1)	0.5 (3)	23.5 (4)	0.0 (0)	0.5 (8)
Glazing agent	0.0 (0)	0.2 (1)	0.5 (3)	23.5 (4)	0.0 (0)	0.5 (8)
Humectant	10.3 (50)	2.4 (11)	3.0 (18)	23.5 (4)	0.0 (0)	5.1 (83)
Packaging gas	0.0 (0)	0.0 (0)	0.3 (2)	0.0 (0)	0.0 (0)	0.1 (2)
Preservative	4.1 (20)	0.4 (2)	1.8 (11)	0.0 (0)	1.5 (1)	2.1 (34)
Propellant	0.0 (0)	0.0 (0)	0.3 (2)	0.0 (0)	0.0 (0)	0.1 (2)
Raising agent	2.5 (12)	0.2 (1)	10.7 (65)	0.0 (0)	0.0 (0)	4.8 (78)
Sequestrant	11.0 (53)	0.7 (3)	1.8 (11)	0.0 (0)	0.0 (0)	4.1 (67)
Stabilizer	12.0 (58)	3.0 (14)	5.3 (32)	23.5 (4)	0.0 (0)	6.6 (108)
Sweetener	0.0 (0)	0.0 (0)	0.0 (0)	11.8 (2)	0.0 (0)	0.1 (2)
Thickener	19.4 (94)	3.0 (14)	12.5 (76)	23.5 (4)	11.8 (8)	12.0 (196)
Any nonpermitted additive	42.8 (207)	10.4 (48)	37.5 (227)	23.5 (4)	11.8 (8)	30.2 (494)

1 Values presented in % (*n*)

## Discussion

Despite growing consumption of commercially processed and packaged foods globally, there has been little research on the degree of processing among CPCF marketed specifically to older IYC and even more limited research on the use of additives in CPCF. Findings from this regional study of CPCF available in 7 Southeast Asian countries reveal concerning levels of ultraprocessing and the use of unnecessary cosmetic and often nonpermitted additives in products marketed as appropriate for older IYC.

Nearly half of all CPCF identified in this study were categorized as UPF according to the Nova classification, with total sugar and sodium contents significantly higher among UPF compared with unprocessed/minimally processed products. Prior research exploring levels of processing among CPCF has also indicated substantial proportions of products to be UPF; however, the prevalence of UPF in the CPCF market varies by context. A study of 3427 CPCF available across 27 European countries found just >1/4 (29%) of products to be ultraprocessed, with higher levels of energy, saturated fat, total sugar, and sodium content among UPF products compared with products with lesser levels of processing [19]. Almost 2/3 (62%) of CPCF available in 2021 in Portugal were UPF, with ultraprocessed products also containing greater energy density and saturated fat than unprocessed/minimally processed CPCF (20). Further, an Australian study assessing CPCF marketed for children aged 12–36 mo reported 85% of products to be UPF, and although sodium content increased with level of processing, total sugar content showed an inverse relationship [39]. The variation in UPF across CPCF markets may be partially explained by variation in the CPCF categories available across geographies; this present Southeast Asia study and prior research indicate that dry/instant cereals, beverages, and snacks/finger foods trend toward higher proportions of UPF than other CPCF categories [19,39].

The substantial proportion of CPCF identified as UPF in this study indicates that IYC diets in the Southeast Asia region are likely becoming more highly processed [40]. The relationship between UPF consumption and adverse health and nutrition outcomes has been noted in research over the last decade, including associations with weight gain, cardiovascular disease, and increased risk of other diet-related noncommunicable diseases [[41], [42], [43], [44], [45]]. Although the literature on the impact of UPF consumption on IYC health outcomes is limited, studies among older children indicate that higher UPF consumption in childhood is associated with excess weight gain and reduced diet quality [11,46]. Given that older IYC are developmentally more vulnerable and have higher nutrient requirements than older children and adults, the substantial presence of UPF in their diets likely holds detrimental implications for older IYC nutritional well-being. Beyond health and nutrition impacts, the globalization and industrialization of the food system and rise of UPF consumption holds environmental implications, including associations with increased greenhouse gas emissions and reduced biodiversity [47,48] and sociocultural impacts on food culture and traditions [49,50] that will negatively affect generations to come.

Despite ∼1/2 of all CPCF being UPF, it must be noted that in equal measure, >1/2 of CPCF identified in this study were not ultraprocessed. Over 1/3 of dry/instant cereals, purees/meals, and condiments were unprocessed/minimally processed, and >1/3 of snacks/finger foods were processed, showing that lower levels of processing are possible across all CPCF categories. Use of UPF for older IYC feeding is higher among caregivers with lower levels of education and household income [51], and one criticism of the argument against UPF has been that regulations against such foods could be discriminatory to populations of lower socioeconomic status who are time-burdened and rely on these foods for convenience [52,53]. Findings from this study indicate that it is possible for manufacturers to develop, produce, and market CPCF that are not reliant on ultraprocessing, but that are still ready-to-eat and packaged conveniently for caregivers of older IYC. If these products are also nutritionally suitable in their composition, appropriately labeled, and in line with global standards to ensure food safety [25,54], such minimally processed CPCF could provide an option for families in need of time-saving options.

Use of additives among products marketed for older IYC in the Southeast Asia region was highly prevalent, with ∼1/2 (48%) of all products containing additives and a median of 3 additives per product. A Bolivian study of CPCF identified the presence of 41 additives across 26 products available in 3 urban centers, with the most common functional classes of additives being antioxidants, colors, and acidity regulators [55]. A study in the United States found that the proportion of household purchases of CPCF containing 4 common food additives (colors, flavors, preservatives, and nonnutritive sweeteners) rose from 29% to 48% between 2001 and 2019, with the mean number of additives per product also increasing from 1.5 to 3.2 [4]. Although the body of evidence is extremely limited, this present study from the Southeast Asia region provides another datapoint revealing the common use of additives in CPCF.

The use of some additives in food products is not inherently a concern for young child health, as many additives are not known to hold negative consequences for health, and their appropriate use can aid food preservation and safety [56]. However, increasing evidence has shown that some types of additives, particularly those used for cosmetic rather than functional purposes, may have negative implications for human health and nutrition outcomes. More than 30% of all CPCF in this Southeast Asian study made use of cosmetic additives to enhance products’ appearance, flavor, or texture. Specifically, 20% and 19% of CPCF contained emulsifiers and color additives, respectively, with a median number of 2 cosmetic additives used among products. The use of certain emulsifiers—additives used to improve products’ texture, appearance, and mouthfeel—has been associated with intestinal inflammation, with deleterious effects on gut microbiota and permeability [15], as well as insulin resistance and the potential increased risk of diabetes [57]. Research among children consuming foods/beverages containing certain color additives has found associations with behavioral outcomes, including hyperactivity and attention-deficit disorders [58].

Almost 1/3 of CPCF assessed in this study contained additives not permitted in Codex standards and guidelines for these products. Codex allowance of any additives, synthetic and natural, in food products is based on safety assessments conducted by the Joint FAO/WHO Expert Committee on Food Additives, which includes a review of all toxicologic research on an additive through mandatory animal testing and available studies among humans [59]. In addition to stipulating the allowance of certain additives in CPCF, Codex also provides maximum levels per 100 g of CPCF product for each permitted additive. These maximum levels are based on acceptable daily intake (ADI) values, defined as the estimated amount of a substance in food, expressed on a body weight basis, that can be safely consumed daily throughout life without risk to the consumer [60,61]. ADIs are developed through extensive hazard characterization, exposure assessment, and risk characterization; however, they are developed for adult populations, and the appropriateness of ADIs for older IYC is not clear [62]. Compared with adults, older IYC have greater dietary intake per kilogram weight and have less varied diets, potentially consuming only several kinds of foods as they enter the complementary feeding period [62,63]. Further, as their organ systems are still maturing, older IYC may be more vulnerable to the health effects of additives [62]. Recent studies further point to the presence of ‘cocktails’ of co-occurring additives in certain UPF product categories [64,65], including cosmetic additives. The accumulative biologic effects and impacts on human health are yet to be fully investigated, and the impact of such groupings specifically on child health is not known. For these reasons, the use of additives in CPCF without any functional necessity should be avoided, and those which are not permitted must not be included as an ingredient of CPCF to ensure child health.

This study is one of very few pieces of research to assess levels of processing among CPCF and to review the types of additives used in these products. The inclusion of products across 7 countries in 1 region provides a comprehensive landscape for the Southeast Asia region. However, limitations should be noted. First, the assessment of processing relied solely on review of label information because it was not possible to assess the actual processing methods applied to products. Although this is a limitation, it is consistent with research using the Nova classification system to identify UPF [12] and the methodology used in this study to search for all possible markers of processing in ingredient lists has been shown to be the most comprehensive for identifying UPF [38]. Second, only the presence and types of additives were identified in products, rather than the quantities of additives. Although assessing levels of additives in CPCF was not within the scope of this study, future research would be valuable to explore if the quantities used are within levels permitted by Codex and in line with national regulations. Third, the primary study from which this dataset was sourced excluded products whose labels were in a language that was not the local language of the country or English. For most countries in the dataset, this resulted in the exclusion of <10 products; however, 215 products in Vietnam were excluded for this reason. As this represents ∼1/2 of the 457 products initially identified in Vietnam, the exclusion of these products prohibited assessment of processing and the presence of additives of a substantial proportion of CPCF available on the market in this country. This presents a substantial limitation of this data set, and it is recommended that future studies ensure translation of all product labels identified. Finally, this data set sampled CPCF available in the capital cities of each country. Although the sampling strategy used to identify products in each city has been shown in prior research to cover most products available nationally [66,67], the products included may not be fully representative of the national level market.

In conclusion, substantial proportion of products marketed for older infants and young children in the Southeast Asia region are ultraprocessed and contain additives, many of which are not permitted by Codex Alimentarius. Considering the growing evidence on the impact of UPF consumption on health and nutrition outcomes, as well as the potentially deleterious effects of certain additives, this study holds concerning implications for the diets of older infants and young children. These findings should alert national governments to the urgent need to both adopt and ensure enforcement of Codex guidance on additives and regulations enacted to encourage lower levels of processing for CPCF.

## Author contributions

The authors’ responsibilities were as follows – AMP, RK, EB, JB: designed the research; AMP, EB: conducted the research; AMP: analyzed data; AMP: wrote the paper; and all authors: provided critical review and approved the final manuscript.

## Conflict of interest statement

The authors report no conflicts of interest. At the time of writing, Roland Kupka and Jessican Blankenship were UNICEF staff members while Alissa M. Pries and Eleonora Bassetti were UNICEF consultants. The opinions and statements in this article are those of the authors and may not reflect official UNICEF policies.

## Funding

This research was funded, in whole or in part, by the Bill & Melinda Gates Foundation to UNICEF through the Regional Initiatives for Sustained Improvements in Nutrition and Growth [grant OPP1179059]. Under the grant conditions of the Foundation, a Creative Commons Attribution 4.0 Generic License has already been assigned to the Author Accepted Manuscript version that might arise from this submission. Phil Baker is supported by an Australian Research Council Future Fellowship Award (project number FT220100690) funded by the Australian Government.

## Data availability

Data described in the manuscript, code book, and analytic code will be made available upon request pending application and approval.

## Appendix A Supplementary data

multimedi component 1 multimedi component 1
