# Supplementary material for: Ultraprocessing and presence of additives in commercially produced complementary foods in seven Southeast Asian countries: a cross-sectional study
Source: Am J Clin Nutr. 2024 May 28;120(2):310–9. doi: 10.1016/j.ajcnut.2024.04.003 (PMC11347792; doi:10.1016/j.ajcnut.2024.04.003)

**Supplementary materials**

**TITLE:** Ultra-processing and presence of additives in commercially produced complementary foods in seven Southeast Asian countries: a cross-sectional study

**AUTHORS**: Alissa M. Pries, Eleonora Bassetti, Jane Badham, Philip Baker, Jessica Blankenship, Elizabeth K. Dunford, Roland Kupka

| Supplemental Table 1. Search terms for additive functional classes, inorganic phosphate additives, and nitrates | |
| --- | --- |
| Emulsifiers | emulsifier 472a "acetic acid ester" acetoglycerides "acetylated monoglyceride" "acetylated diglyceride" "fatty acid ester" 1422 "acetylated distarch adipate" 1414 "acetylated distarch phosphate" 1451 "acetylated oxidized starch" 1401 "starch, acid-treated" 1401 "acid-treated starch" "acid treated starch" 406 agar "agar-agar" "japan agar" "layor carang" 400 "alginic acid" 1402 "alkaline treated starch" "starch, alkaline treated" 403 "ammonium alginate" 452v "ammonium polyphosphate" 442 "ammonium phosphatide" "ammonium salt of phosphatidic acid" 901 beeswax 1403 "bleached starch" "starch, bleached" 542 "bone phosphate" 450vii "acid calcium pyrophosphate" "monocalcium di hydrogen pyrophosphate" "calcium dihydrogen diphosphate" 452iv "calcium polyphosphate" 482i "calcium stearoyl lactylate" 902 "candelilla wax" 410 "carob bean gum" "algaroba" "carob gum" "locust bean gum" 407 "carrageenan" "danish agar" "eucheuman" "furcellaran agar" hypnean "irish moss gelose" iridophycan 427 "cassia gum" 1503 "castor oil" "ricinus oil" 472c citrem "citric acid ester" "citroglyceride" 1400 "dextrins, roasted starch" 472e datem "diacetyltartaric acid ester" "diacetyltartaric and fatty acid ester of glycerol" 450vi "dicalcium diphosphate" "calcium pyrophosphate" "dicalcium pyrophosphate" 480 "dioctyl sodium sulfosuccinate" "docusate sodium" dss 340ii "dipotassium hydrogen phosphate" 450i "disodium diphosphate" 339ii "disodium hydrogen phosphate" "dibasic sodium phosphate" "disodium acid phosphate" "disodium hydrogen monophosphate" "disodium hydrogen phosphate" "disodium phosphate" "secondary sodium phosphate" 1412 "distarch phosphate" 467 "ethyl hydroxyethyl cellulose" 445iii "glycerol ester of wood rosin" "ester gum" 412 "guar gum" "guar flour" "gum cyamopsis" 414 "gum arabic" "acacia gum" "arabic gum" 419 "gum ghatti" 463 "hydroxypropyl cellulose" "cellulose hydroxypropyl ether" "modified cellulose" 1442 "hydroxypropyl distarch phosphate" 464 "hydroxypropyl methyl cellulose" 1440 "hydroxypropyl starch" 416 "karaya gum" "gum karaya "gum sterculia" "kadaya" "karaya" "katilo" "kullo" "kutterra" "sterculia" 425 "konjac flour" "konjac" "konjac mannen" "konnyaku" "konnyaleu" 472b "lactic acid ester" "lactoglyceride" 966 "lactitol" "lactit" "lactobiosit" "lactositol" 322i "lecithin" "phosphatide" "phospholipid" 322ii "lecithin, partially hydrolysed" 470iii "magnesium stearate" 965i "maltitol" "d-maltitol" "dried maltitol syrup" "hydrogenated glucose syrup" "hydrogenated high maltose-content glucose syrup" "hydrogenated maltose" "maltitol syrup powder" 965ii "maltitol syrup" 461 "methyl cellulose" "cellulose methyl ether" "methyl ether of cellulose" 465 "methyl ethyl cellulose" MEC "methyl ethyl ether of cellulose" 460i "microcrystalline cellulose" "cellulose gel" 471 "glyceryl monooleate" "glyceryl monoplamitate" "glyceryl monostearate" GMS monoolein monopalmitin monostearin 1410 "monostarch phosphate" 423 "octenyl succinic acid" "modified gum arabic" 1401 "oxidized starch" 440 pectin 451ii "pentapotassium triphosphate" 451i "pentasodium triphosphate" "pentasodium tripolyphosphate" "sodium triphosphate" triphosphate "sodium tripolyphosphate" 1413 "phosphated distarch phosphate" 900a polydimethylsiloxane dimethylpolysiloxane "dimethylsilicone fluid" "dimethylsilicone oil" 1521 "polyethylene glycol" macrogol peg 475 "glycerin fatty acid ester" "polyglycerol fatty acid ester" 476 "glyceran esters of condensed castor oil fatty acid" "polyglycerol esters of interesterified ricinoleic acid" "polyglycerol esters of polycondensed fatty acids from castor oil" 432 "polysorbate 20" 433 "polysorbate 80" 434 "polysorbate 40" 435 "polysorbate 60" 436 "polysorbate 65" 431 "polyoxyl (40) stearate" "polyoxyethylene (40) monostearate" "polyoxyethylene (40) stearate" 430 "polyoxyethylene (8) stearate" "polyoxyl (8) stearate" 1201 polyvinylpyrrolidone povidone pvp 402 "potassium alginate" 340i "potassium dihydrogen phosphate" "monobasic potassium phosphate" "monopotassium dihydrogen monophosphate" "monopotassium dihydrogen orthophosphate" "monopotassium monophosphate" "potassium acid phosphate" "potassium biphosphate" "potassium dihydrogen phosphate" 326 "potassium lactate" 452ii "potassium polyphosphate" "potassium metaphosphate" 460ii "powdered cellulose" 407a "processed eucheuma seaweed" "png-carrageenan" "semi-refined carrageenan" 1520 "propylene glycol" "methyl glycol" propanediol 405 "propylene glycol alginate" "hydroxypropyl alginate" "propane 1,2-diol alginate" "1,2-propane-diol ester of alginic acid" 477 "Propane-1,2-diol esters of fatty acids" "propylene glycol esters of fatty acids" 999ii "quillaia extract type 2" 999i "quillaia extract type 1" 470i "salts of myristic, palmitic and stearic acids with ammonia, calcium, potassium and sodium" 470ii "salts of oleic acid with calcium, potassium and sodium" 401 "sodium alginate" 541i "sodium aluminium phosphate, acidic" SALP 541ii "sodium aluminium phosphate, basic" kasal 452iii "sodium calcium polyphosphate" 466 "sodium carboxymethyl cellulose" "cellulose gum" cmc "na cmc" "sodium cellulose glycolate" "sodium cmc" 331i "sodium dihydrogen citrate" "monosodium citrate" "sodium citrate monobasic" 339i "sodium dihydrogen phosphate" "monobasic sodium phosphate" "monosodium dihydrogen monophosphate" "monosodium dihydrogen orthophosphate" "monosodium monophosphate" "sodium acid phosphate" "sodium biphosphate" "sodium dihydrogen phosphate" 325 "sodium lactate" 452i "sodium polyphosphate" "graham's salt" "sodium hexametaphosphate" "sodium polyphosphate, glassy" "sodium tetrapolyphosphate" 481i "sodium stearoyl lactylate" "sodium stearoyl-2-lactylate" "sodium stearoyl lactate" 493 "sorbitan monolaurate" "sorbitan laurate" 494 "sorbitan monooleate" 495 "sorbitan monopalmitate" 491 "sorbitan monostearate" 492 "sorbitan tristearate" 1420 "starch acetate" 1450 "starch sodium octenyl succinate" 1405 "starch, enzyme treated" "starches, enzyme treated" 484 "stearyl citrate" 474 "sucroglycerides" 473a "sucrose oligoesters, type i and type ii" 444 "sucrose acetate isobutyrate" saib 473 "sucrose fatty acid esters" "sucrose esters of fatty acids" 181 "tannic acid" "gallotannic acid" "tannins" 450v "tetrapotassium diphosphate" "potassium pyrophosphate" "tetrapotassium pyrophosphate" 450iii "tetrasodium diphosphate" "sodium pyrophosphate" "tetrasodium pyrophosphate" 479 "thermally oxidized soya bean oil interacted with mono- and diglycerides of fatty acids" tosom 413 "tragacanth gum" 1518 triacetin 341iii "tricalcium phosphate" "calcium phosphate, tribasic" "precipitated calcium phosphate" "tricalcium phosphate" 1505 "triethyl citrate" "ethyl citrate" 340iii "tripotassium phosphate" "potassium phosphate" "tribasic potassium phosphate" "tripotassium phosphate" 331iii "trisodium citrate" "sodium citrate" 450ii "trisodium diphosphate" "acid trisodium pyrophosphate" "trisodium monohydrogen diphosphate" 339iii "trisodium phosphate" "sodium phosphate" "tribasic sodium phosphate" "trisodium phosphate" 415 "xanthan gum" 967 xylitol |
| Flavour enhancers | 950 951 "flavour enhancer" "flavor enhancer" "acesulfame potassium" "acesulfame k" 969 advantame aspartame apm "aspartyl phenylalanine methyl ester" 1101iii bromelain 629 "calcium 5'-guanylate""calcium guanylate" 633 "calcium 5'-inosinate" "calcium inosinate" 634 "calcium 5'-ribonucleotides" "calcium ribonucleotides" 623 "calcium di-L-glutamate" "calcium glutamate" 628 "dipotassium 5'-guanylate" "dipotassium guanosine-5'-monophosphate" "potassium 5'-guanylate" "potassium guanylate" 627 "disodium 5'-guanylate" "disodium guanosine-5'-monophosphate" "sodium 5'-guanylate" "sodium guanylate" 631 "disodium 5'-inosinate" "disodium inosine-5'-monophosphate" "sodium 5'-inosinate" "sodium inosinate" 635 "disodium 5'-ribonucleotides" "sodium 5'-ribonucleotides" "sodium ribonucleotides" 968 erythritol erythrite "meso-erythritol" "tetrahydroxybutane" 637 "ethyl maltol" 620 "glutamic acid, l(+)-" "l-alpha-aminoglutaric acid" "l-2-amino-pentanedioic acid" "glutamic acid" "l-glutamic acid" "l-(+)-glutamic acid" 626 "guanylic acid, 5'-" gmp "guanosine-5'-monophosphoric acid" "guanylic acid" 630 "inosinic acid, 5'-" imp "inosinic acid" 953 isomalt "hydrogenated isomaltulose" isomaltitol 1104 lipases 625 "magnesium di-l-glutamate" "magnesium glutamate" 580 "magnesium gluconate" 518 "magnesium sulfate" 636 maltol 624 "monoammonium l-glutamate" "ammonium glutamate" 622 "monopotassium l-glutamate" mpg "potassium glutamate" 621 "monosodium l-glutamate" "monosodium glutamate" "sodium glutamate" 961 neotame 1101ii papain 632 "potassium 5'-inosinate" "dipotassium inosine-5'-monophosphate" "potassium 5'-inosinate" "potassium inosinate" 508 "potassium chloride" sylvine sylvite 1101i "protease from aspergillus orizae var" 955 sucralose trichlorogalactosucrose 334 "tartaric acid" 957 thaumatin |
| Colours | 160cii color colour "paprika extract" 129 "allura red" "no.16035" "red 17" "red no.40" 123 amaranth "no. 16185" "red 9" "naphtol rot s" 160bi 160bii "annatto extract" 122 azorubine carmoisine "no. 14720" "red 3" 162 "beet red" "beetroot red" 151 "brilliant black" "black bn" "black pn" "no. 28440" "black 1" 133 "brilliant blue" "no. 42900" "blue 2" "blue no.1" 155 "brown ht" "no. 20285" "brown 3" 170i "calcium carbonate" "chalk" 161g canthaxanthin "no. 40850" "orange 8" 150a "caramel i" "caustic caramel" "plain caramel" 150b "caramel ii" "caustic sulfite caramel" 150c "caramel iii" "ammonia caramel" 150d "caramel iv" "sulfite ammonia caramel" 120 carmine carmines "no. 75470" "red 4" 160e "carotenal, beta-apo-8" "orange 6" 160aiv "β-carotene-rich extract from dunaliella salina" 160aiii "beta-carotenes, blakeslea trispora" 160ai "beta-carotenes, synthetic" 160aii "beta-carotenes, vegetable" "carotenes-natural" "orange 5" "mixed carotenes" "natural beta-carotene" 160f "carotenoic acid" "orange 7" "ethyl ester" 141ii "chlorophyllin copper complexes" "no. 75810" "potassium copper chlorophyllin" "sodium copper chlorophyllin" 140 "chlorophyll" "no. 75810" "green 3" "magnesium chlorophyll" "magnesium phaeophytin" 141i "copper chlorophyll" "copper phaeophytin" 100i curcumin "yellow 3" "turmeric yellow" "kurkum" "diferuloymethane" 127 erythrosine "no. 45430" "red 14" "red no. 3" 143 "fast green" "green 3" "no. 42053" "green no. 3" 163ii "grape skin extract" eno enociania 132 indigotine "blue 1" "no. 73015" "blue no. 2" "indigo carmine" 172i "iron oxide, black" "black 11" "no. 77499" 172ii "iron oxide, red" "red 101" "red 102" "no. 77491" 172iii "iron oxide, yellow" "yellow 42" "yellow 43" "no. 77492" 161biii "lutein esters from tagetes erecta" 161bi "lutein from tagetes erecta" 160diii "lycopene, blakeslea trispora" 160di "lycopene, synthetic" 160dii "lycopene, tomato" 124 "ponceau 4r" "no. 16255" "red 7" "red a" "cochineal red a" "new coccine" 104 "quinoline yellow" "no. 47005" "yellow 13" 101ii "riboflavin 5'-phosphate sodium" "vitamin b2 ester monosodium salt" 101iii "riboflavin from bacillus subtilis" 101i "riboflavin, synthetic" "riboflavin 5'-phosphate ester monosodium salt" "vitamin b2 phosphate ester monosodium salt" 110 "sunset yellow" "no. 15985" "yellow 3" "yellow no. 6" "crelborange s" 181 "tannic acid" "tannins (food grade)" "gallotannic acid" 102 tartrazine "no. 19140" "yellow 4" "yellow no. 5" 171 "titanium dioxide" "no. 77891" "white 6" 161hi "zeaxanthin, synthetic" |
| Emulsifying salts | 452v "emulsifying salts" "emulsifying salt" "ammonium polyphosphate" 450vii "calcium dihydrogen diphosphate" "acid calcium pyrophosphate" "monocalcium dihydrogen pyrophosphate" 341i "calcium dihydrogen phosphate" "acid calcium phosphate" "calcium biphosphate" "calcium dihydrogen phosphate" "calcium dihydrogen tetraoxophosphate" "monobasic calcium phosphate" "monocalcium orthophosphate" "monocalcium phosphate" 341ii "calcium hydrogen phosphate" "calcium hydrogen monophosphate" "calcium hydrogen orthophosphate" "calcium hydrogen phosphate" "dibasic calcium phosphate" "dicalcium phophate" "secondary calcium phosphate" 327 "calcium lactate" 452iv "calcium polyphosphate" 450vi "dicalcium diphosphate" "calcium pyrophosphate" "dicalcium pyrophosphate" 450i "disodium diphosphate" "acid sodium pyrophosphate" "disodium dihydrogen diphosphate" "disodium dihydrogen pyrophosphate" "disodium pyrophosphate" 339ii "disodium hydrogen phosphate" "dibasic sodium phosphate" "disodium acid phosphate" "disodium hydrogen monophosphate" "disodium hydrogen phosphate" "disodium phosphate" "secondary sodium phosphate" 343i "magnesium dihydrogen phosphate" "acid magnesium phosphate" "magnesium biphosphate" "magnesium dihydrogen phosphate" "magnesium phosphate mono basic" "monomagnesium orthophosphate" 343ii "magnesium hydrogen phosphate" "dibasic magnesium phosphate" "dimagnesium phosphate" "magnesium hydrogen phosphate" "magnesium phosphate, dibasic" "secondary magnesium phosphate" 451ii "pentapotassium triphosphate" "pentapotassium tripolyphosphate" "potassium triphosphate" "potassium tripolyphosphate" 451i "pentasodium triphosphate" "pentasodium tripolyphosphate" "sodium triphosphate" "sodium tripolyphosphate" triphosphate 332i "potassium dihydrogen citrate" "monopotassium citrate" "potassium citrate monobasic" 452ii "potassium polyphosphate" "potassium metaphosphate" 337 "potassium sodium l(+)-tartrate" "potassium sodium dextro-tartrate" "rochelle salt" "seignette salt" 335ii "sodium l(+)-tartrate" "sodium dextro-tartrate" "sodium tartrate, l (+)-" 541i "sodium aluminium phosphate, acidic" salp 541ii "sodium aluminium phosphate, basic" kasal 500i "sodium carbonate" "soda ash" 331i "sodium dihydrogen citrate" "monosodium citrate" "sodium citrate monobasic" 339i "sodium dihydrogen phosphate" "monobasic sodium phosphate" "monosodium dihydrogen monophosphate" "monosodium dihydrogen orthophosphate" "monosodium monophosphate" "sodium acid phosphate" "sodium biphosphate" "sodium dihydrogen phosphate" 325 "sodium lactate" 452i "sodium polyphosphate" "graham's salt" "sodium hexametaphosphate" "sodium polyphosphate, glassy" "sodium tetrapolyphosphate" 437 "tamarind seed polysaccharide" 450v "tetrapotassium diphosphate" "potassium pyrophosphate" "tetrapotassium pyrophosphate" 450iii "tetrasodium diphosphate" "sodium pyrophosphate" "tetrasodium pyrophosphate" 333iii "tricalcium citrate" 341iii "tricalcium phosphate" "calcium phosphate, tribasic" "precipitated calcium phosphate" "tricalcium phosphate" 332ii "tripotassium citrate" "potassium citrate" 340iii "tripotassium phosphate" "potassium phosphate" "tribasic potassium phosphate" "tripotassium phosphate" 331iii "trisodium citrate" "sodium citrate" 450ii "trisodium diphosphate" "acid trisodium pyrophosphate" "trisodium monohydrogen diphosphate" |
| Sweeteners | 950 "acesulfame potassium" "acesulfame k" 969 advantame 956 alitame 951 aspartame apm "aspartyl phenylalanine methyl ester" 962 "aspartame-acesulfame salt" 952ii "calcium cyclamate" 954ii "calcium saccharin" 952i "cyclamic acid" "cyclohexylsulfamic acid" 960c "enzymatically produced steviol glycosides" 968 erythritol erythrite meso-erythritol tetrahydroxybutane 960d "glucosylated steviol glycosides" 953 "hydrogenated isomaltulose" isomalt isomaltitol 966 lactitol lactit lactobiosit lactositol 965i maltitol "d-maltitol" "dried maltitol syrup" "hydrogenated glucose syrup" "hydrogenated high maltose-content glucose syrup" "hydrogenated maltose" "maltitol syrup powder" 965ii "maltitol syrup" 421 "mannitol mannite d-mannitol" 961 neotame 964 "polyglycitol syrup" 954iii "potassium saccharin" 954i saccharin 952iv "sodium cyclamate" 954iv "sodium saccharin" 420i sorbitol "d-glucitol" "d-glucitol syrup" sorbit "d-sorbitol" sorbol 420ii "sorbitol syrup" 960a "steviol glycosides from stevia rebaudiana bertoni" "steviol glycosides from stevia" 960b "steviol glycosides from fermentation" 955 sucralose "4,1',6'-trichlorogalactosucrose" trichlorogalactosucrose 957 thaumatin 967 xylitol |
| Thickeners | 1422 thickener thickeners "acetylated distarch adipate" 1414 "acetylated distarch phosphate" 1451 "acetylated oxidized starch" 1401 "acid-treated starch" "starch, acid-Treated" 406 agar "agar-agar" "bengal, ceylon, chinese or japanese isinglass" "gelose" "japan agar" "layor carang" 400 "alginic acid" 1402 "alkaline treated starch" "starch, alkaline treated" 403 "ammonium alginate" 342i "ammonium dihydrogen phosphate" "acid ammonium phosphate" "ammonium dihydrogen orthophosphate" "ammonium dihydrogen phosphate" "ammonium dihydrogen tetraoxophosphate" "monoammonium monophosphate" "monoammonium phosphate" "monobasic ammonium phosphate" "primary ammonium phosphate" 341i "calcium dihydrogen phosphate" "acid calcium phosphate" "calcium biphosphate" "calcium dihydrogen phosphate" "calcium dihydrogen tetraoxophosphate" "monobasic calcium phosphate" "monocalcium orthophosphate" "monocalcium phosphate" 341ii "calcium hydrogen phosphate" "calcium hydrogen monophosphate" "calcium hydrogen orthophosphate" "calcium hydrogen phosphate" "dibasic calcium phosphate" "dicalcium phophate" "secondary calcium phosphate" 327 "calcium lactate" 452iv "calcium polyphosphate" 902 "candelilla wax" 410 "carob bean gum" algaroba "carob gum" "locust bean gum" 407 "carrageenan" "danish agar" "eucheuman" "furcellaran agar" hypnean "irish moss gelose" iridophycan 427 "cassia gum" 468 "cross-linked sodium carboxymethyl cellulose" "cross-linked-cellulose gum" 424 curdlan "beta-1,3-glucan" 457 "cyclodextrin, alpha-" 459 "cyclodextrin, beta-" bcd betacd "cyclodextrin b" "beta-schardinger dextrin" 458 "cyclodextrin, gamma-" 1400 "dextrins, roasted starch" 342ii "diammonium hydrogen phosphate" "ammonium dihydrogen orthophosphate" "ammonium dihydrogen phosphate" "ammonium dihydrogen tetraoxophosphate" "diammonium hydrogenorthophosphate" "diammonium hydrogen phosphate" "diammonium hydrogentetraoxophosphate" "diammonium phosphate" "dibasic ammonium phosphate" 450vi "dicalcium diphosphate" "calcium pyrophosphate" "dicalcium pyrophosphate" 340ii "dipotassium hydrogen phosphate" "dibasic potassium phosphate" "dipotassium acid phosphate" "dipotassium hydrogen monophosphate" "dipotassium hydrogen orthophosphate" "dipotassium hydrogen phosphate" "dipotassium monophosphate" "dipotassium phosphate" "secondary potassium phosphate" 450i "disodium diphosphate" "acid sodium pyrophosphate" "disodium dihydrogen diphosphate" "disodium dihydrogen pyrophosphate" "disodium pyrophosphate" 339ii "disodium hydrogen phosphate" "dibasic sodium phosphate" "disodium acid phosphate" "disodium hydrogen monophosphate" "disodium hydrogen phosphate" "disodium phosphate" "secondary sodium phosphate" 1412 "distarch phosphate" 462 "ethyl cellulose" 467 "ethyl hydroxyethyl cellulose" 418 "gellan gum" 422 "glycerol" "glycerin" 412 "guar gum" "guar flour" "gum cyamopsis" 414 "gum arabic" "acacia gum" "arabic gum" 419 "gum ghatti" 463 "hydroxypropyl cellulose" "cellulose hydroxypropyl ether" "modified cellulose" 1442 "hydroxypropyl distarch phosphate" 464 "hydroxypropyl methyl cellulose" 1440 "hydroxypropyl starch" 953 isomalt "hydrogenated isomaltulose" isomaltitol 416 "karaya gum" "gum karaya" "gum sterculia" "kadaya" "karaya" "katilo" "kullo" "kutterra" "sterculia" 425 "konjac flour" "konjac mannen" konjac konnyaku konnyaleu 966 lactitol lactit lactobiosit lactositol 343i "magnesium dihydrogen phosphate" "acid magnesium phosphate" "magnesium biphosphate" "magnesium dihydrogen phosphate" "magnesium phosphate mono basic" "monomagnesium orthophosphate" 343ii "magnesium hydrogen phosphate" "dibasic magnesium phosphate" "dimagnesium phosphate" "magnesium hydrogen phosphate" "magnesium phosphate, dibasic" "secondary magnesium phosphate" 470iii "magnesium stearate" 965i maltitol "d-maltitol" "dried maltitol syrup" "hydrogenated glucose syrup" "hydrogenated high maltose-content glucose syrup" "hydrogenated maltose" "maltitol syrup powder" 965ii "maltitol syrup" 421 mannitol mannite "d-mannitol" 461 "methyl cellulose" "cellulose methyl ether" "methyl ether of cellulose" 465 mec "methyl ethyl cellulose" "methyl ethyl ether of cellulose" 460i "microcrystalline cellulose" "cellulose gel" 1410 "monostarch phosphate" 1404 "oxidized starch" 440 pectin 451ii "pentapotassium triphosphate" "pentapotassium tripolyphosphate" "potassium triphosphate" "potassium tripolyphosphate" 451i "pentasodium triphosphate" "pentasodium tripolyphosphate" "sodium triphosphate" "sodium tripolyphosphate" "triphosphate" 1413 "phosphated distarch phosphate" 1200 polydextroses "modified polydextroses" 1521 "polyethylene glycol" macrogol peg 1203 "polyvinyl alcohol" pvoh "vinyl alcohol polymer" 1201 polyvinylpyrrolidone povidone pvp 402 "potassium alginate" 508 "potassium chloride" sylvine sylvite 340i "potassium dihydrogen phosphate" "monobasic potassium phosphate" "monopotassium dihydrogen monophosphate" "monopotassium dihydrogen orthophosphate" "monopotassium monophosphate" "potassium acid phosphate" "potassium biphosphate" "potassium dihydrogen phosphate" 452ii "potassium polyphosphate" "potassium metaphosphate" 460ii "powdered cellulose" 407a "processed eucheuma seaweed" "png-carrageenan" "semi-refined carrageenan" 405 "propylene glycol alginate" "hydroxypropyl alginate" "propane 1,2-diol alginate" "1,2-propane-diol ester of alginic acid" 1204 pullulan 401 sodium alginate 541i "sodium aluminium phosphate, acidic" salp 541ii "sodium aluminium phosphate, basic" kasal 500i "sodium carbonate" "soda ash" 466 "sodium carboxymethyl cellulose" "cellulose gum" cmc "na cmc" "sodium cellulose glycolate" "sodium cmc" 469 "sodium carboxymethyl cellulose, enzymatically hydrolysed" "cellulose gum, enzymatically hydrolyzed" 339i "sodium dihydrogen phosphate" "monobasic sodium phosphate" "monosodium dihydrogen monophosphate" "monosodium dihydrogen orthophosphate" "monosodium monophosphate" "sodium acid phosphate" "sodium biphosphate" "sodium dihydrogen phosphate" 576 "sodium gluconate" "sodium d-gluconate" 500ii "sodium hydrogen carbonate" "baking soda" "bicarbonate of soda" "sodium bicarbonate" 325 "sodium lactate" 452i "sodium polyphosphate" "graham's salt" "sodium hexametaphosphate" "sodium polyphosphate, glassy" "sodium tetrapolyphosphate" 420i sorbitol "d-glucitol" "d-glucitol syrup" sorbit "d-sorbitol" sorbol 420ii "sorbitol syrup" 1420 "starch acetate" "starch acetate esterified with acetic anhydride" "starch acetate esterified with vinyl acetate" 1450 "starch sodium octenyl succinate" 1405 "starches, enzyme treated" "starch, enzyme treated" 553iii talc talcum 437 "tamarind seed polysaccharide" 181 "tannic acid (tannins)" "gallotannic acid" "tannins (food grade)" 417 "tara gum" "peruvian carob" 450v "tetrapotassium diphosphate" "potassium pyrophosphate" "tetrapotassium pyrophosphate" 450iii "tetrasodium diphosphate" "sodium pyrophosphate" "tetrasodium pyrophosphate" 413 "tragacanth gum" 341iii "tricalcium phosphate" "calcium phosphate, tribasic" "precipitated calcium phosphate" "tricalcium phosphate" 343iii "trimagnesium phosphate" "magnesium phosphate, tribasic" "tertiary magnesium phosphate" "trimagnesium phosphate" 340iii "tripotassium phosphate" "potassium phosphate" "tribasic potassium phosphate" "tripotassium phosphate" 450ii "trisodium diphosphate" "acid trisodium pyrophosphate" "trisodium monohydrogen diphosphate" 339iii "trisodium phosphate" "sodium phosphate" "tribasic sodium phosphate" "trisodium phosphate" 415 "xanthan gum" 967 xylitol |
| Antifoaming agents | 404 "antifoaming agent" "calcium alginate" 905ci "microcrystalline wax" "hydrocarbon waxes" "petroleum wax" 905d "mineral oil, high viscosity" "food grade mineral oil" "liquid paraffin" "liquid petrolatum" "white mineral oil" 471 "mono- and di-glycerides of fatty acids" "glyceryl monooleate" "glyceryl monoplamitate" "glyceryl monostearate" gms monoolein monopalmitin monostearin 900a polydimethylsiloxane dimethylpolysiloxane "dimethylsilicone fluid" "dimethylsilicone oil" "poly(dimethylsiloxane)" 1521 "polyethylene glycol" macrogol peg 551 "silicon dioxide, amorphous" silica |
| Bulking agents | 406 "bulking agent" agar "agar-agar" "bengal, ceylon, chinese or japanese isinglass" "gelose" "japan agar" "layor carang" 400 "alginic acid" 403 "ammonium alginate" 404 "calcium alginate" 903 "carnauba wax" 407 "carrageenan" "danish agar" "eucheuman" "furcellaran agar" hypnean "irish moss gelose" iridophycan 462 "ethyl cellulose" 414 "gum arabic" "acacia gum" "arabic gum" 464 "hydroxypropyl methyl cellulose" 953 isomalt "hydrogenated Isomaltulose" isomaltitol 965i maltitol "d-maltitol" "dried maltitol syrup" "hydrogenated glucose syrup" "hydrogenated high maltose-content glucose syrup" "hydrogenated maltose" "maltitol syrup powder" 965ii "maltitol syrup" 421 mannitol mannite d-mannitol 461 "methyl cellulose" "cellulose methyl ether" "methyl ether of cellulose" 460i "microcrystalline cellulose" "cellulose gel" 1200 polydextroses "modified polydextroses" 402 "potassium alginate" 460ii "powdered cellulose" 407a "processed eucheuma seaweed" "png-carrageenan" "semi-refined carrageenan" 405 "propylene glycol alginate" "hydroxypropyl alginate" "propane 1,2-diol alginate" "1,2-propane-diol ester of alginic acid" 401 "sodium alginate" 466 "sodium carboxymethyl cellulose" "cellulose gum" cmc "na cmc" "sodium cellulose glycolate" "sodium cmc" 325 "sodium lactate" 420i sorbitol "d-glucitol" "d-glucitol syrup" sorbit "d-sorbitol" sorbol 420ii "sorbitol syrup" |
| Carbonating agents | 290 "carbon dioxide" "carbonic acid anhydride" "dry ice" |
| Foaming agents | 400 "alginic acid" 403 "ammonium alginate" 404 "calcium alginate" 482i "calcium stearoyl lactylate" "calcium stearoyl-2-lactylate" "calcium stearoyl lactate" 290 "carbon dioxide" "carbonic acid anhydride" "dry ice" 463 "hydroxypropyl cellulose" "cellulose hydroxypropyl ether" "modified cellulose" 465 "methyl ethyl cellulose" "methyl ethyl ether of cellulose" 460i "microcrystalline cellulose" "cellulose gel" 941 nitrogen 942 "nitrous oxide" "dinitrogen monoxide" "nitrogen oxide" 402 "potassium alginate" 405 "propylene glycol alginate" "hydroxypropyl alginate" "propane 1,2-diol alginate" "1,2-propane-diol ester of alginic acid" 999ii "quillaia extract type 2" 999i "quillaia extract type" "bois de panama" "panama bark extract" "quillai extract" "quillaja extract" "quillay bark extract" "soapbark extract" 401 "sodium alginate" 481i "sodium stearoyl lactylate" "sodium stearoyl-2-lactylate" "sodium stearoyl lactate" 473 "sucrose esters of fatty acids" "sucrose fatty acid esters" 415 "xanthan gum" |
| Gelling agents | 406 "gelling agent" agar "agar-agar" "bengal, ceylon, chinese or japanese isinglass" "gelose" "japan agar" "layor carang" 400 "alginic acid" 403 "ammonium alginate" 404 "calcium alginate" 407 "carrageenan" "danish agar" "eucheuman" "furcellaran agar" hypnean "irish moss gelose" iridophycan 427 "cassia gum" 424 "curdlan" "beta-1,3-glucan" 418 "gellan gum" 425 "konjac flour" "konjac mannen" konjac konnyaku konnyaleu 440 pectin 402 "potassium alginate" 407a "processed eucheuma seaweed" "png-carrageenan" "semi-refined carrageenan" 405 "propylene glycol alginate" "hydroxypropyl alginate" "propane 1,2-diol alginate" "1,2-propane-diol ester of alginic acid" 401 "sodium alginate" 466 "sodium carboxymethyl cellulose" "cellulose gum" "cmc" "na cmc" "sodium cellulose glycolate" "sodium cmc" 437 "tamarind seed polysaccharide" 417 "tara gum" "peruvian carob" |
| Glazing agents | 406 "glazing agent" agar "agar-agar" "bengal, ceylon, chinese or japanese isinglass" "gelose" "japan agar" "layor carang" 400 "alginic acid" 403 "ammonium alginate" 901 beeswax 404 "calcium alginate" 902 "candelilla wax" 903 "carnauba wax" 407 "carrageenan" "danish agar" "eucheuman" "furcellaran agar" hypnean "irish moss gelose" iridophycan 1503 "castor oil" "ricinus oil" 462 "ethyl cellulose" 414 "gum arabic" "acacia gum" "arabic gum" 907 "hydrogenated poly-1-decenes" "hydrogenated polydec-1-ene" "hydrogenated poly-α-olefin" 463 "hydroxypropyl cellulose" "cellulose hydroxypropyl ether" "modified cellulose" 464 "hydroxypropyl methyl cellulose" 953 isomalt "hydrogenated isomaltulose" isomaltitol 425 "konjac flour" "konjac mannen" konjac konnyaku konnyaleu 1205 "methacrylate copolymer, basic" bmc 461 "methyl cellulose" "cellulose methyl ether" "methyl ether of cellulose" 460i "microcrystalline cellulose" "cellulose gel" 905ci "microcrystalline wax" "hydrocarbon waxes" "petroleum wax" 905d "mineral oil, high viscosity" "food grade mineral oil" "liquid paraffin" "liquid petrolatum" "white mineral oil" 905e "mineral oil, medium viscosity" 471 "mono- and di-glycerides of fatty acids" "glyceryl monooleate" "glyceryl monoplamitate" "glyceryl monostearate" gms monoolein monopalmitin monostearin 440 pectin 1200 polydextroses "modified polydextroses" 1521 "polyethylene glycol" "macrogol" peg 1203 "polyvinyl alcohol" pvoh "vinyl alcohol polymer" 1209 "polyvinyl alcohol (pva) –polyethylene glycol (peg) graft copolymer" 1201 polyvinylpyrrolidone povidone pvp 402 "potassium alginate" 460ii "powdered cellulose" 407a "processed eucheuma seaweed" "png-carrageenan" "semi-refined carrageenan" 1520 "propylene glycol" "methyl glycol" "propanediol" 1204 pullulan 904 "shellac, bleached" 401 "sodium alginate" 466 "sodium carboxymethyl cellulose" "cellulose gum" cmc "na cmc" "sodium cellulose glycolate" "sodium cmc" 473a "sucrose oligoesters, type i and type ii" 473 "sucrose esters of fatty acids" "sucrose fatty acid esters" 553iii talc talcum |
| Acidity regulators | 260 "acidity regulator" "acetic acid" 355 "adipic acid" 523 "aluminium ammonium sulfate" "ammonium alum" 503i "ammonium carbonate" 342i "ammonium dihydrogen phosphate" "acid ammonium phosphate" "ammonium dihydrogen orthophosphate" "ammonium dihydrogen phosphate" "ammonium dihydrogen tetraoxophosphate" "monoammonium monophosphate" "monoammonium phosphate" "monobasic ammonium phosphate" "primary ammonium phosphate" 503ii "ammonium hydrogen carbonate" "ammonium bicarbonate" 527 "ammonium hydroxide" 452v "ammonium polyphosphate" 300 "ascorbic acid, l-" 263 "calcium acetate" 170i "calcium carbonate" chalk 450vii "calcium dihydrogen diphosphate" "acid calcium pyrophosphate" "monocalcium dihydrogen pyrophosphate" 341i "calcium dihydrogen phosphate" "acid calcium phosphate" "calcium biphosphate" "calcium dihydrogen phosphate" "calcium dihydrogen tetraoxophosphate" "monobasic calcium phosphate" "monocalcium orthophosphate" "monocalcium phosphate" 578 "calcium gluconate" "calcium di-d-gluconate monohydrate" "calcium di-gluconate" 341ii "calcium hydrogen phosphate" "calcium hydrogen monophosphate" "calcium hydrogen orthophosphate" "calcium hydrogen phosphate" "dibasic calcium phosphate" "dicalcium phophate" "secondary calcium phosphate" 526 "calcium hydroxide" "slaked lime" 327 "calcium lactate" 352ii "calcium malate, d, l-" "d,l-monocalcium malate" "monocalcium malate, d, l-" 529 "calcium oxide" 452iv "calcium polyphosphate" 516 "calcium sulfate" 903 "carnauba wax" 330 "citric acid" 342ii "diammonium hydrogen phosphate" "ammonium dihydrogen orthophosphate" "ammonium dihydrogen phosphate" "ammonium dihydrogen tetraoxophosphate" "diammonium hydrogenorthophosphate" "diammonium hydrogen phosphate" "diammonium hydrogentetraoxophosphate" "diammonium phosphate" "dibasic ammonium phosphate" 450vi "dicalcium diphosphate" "calcium pyrophosphate" "dicalcium pyrophosphate" 340ii "dipotassium hydrogen phosphate" "dibasic potassium phosphate" "dipotassium acid phosphate" "dipotassium hydrogen monophosphate" "dipotassium hydrogen orthophosphate" "dipotassium hydrogen phosphate" "dipotassium monophosphate" "dipotassium phosphate" "secondary potassium phosphate" 450i "disodium diphosphate" "acid sodium pyrophosphate" "disodium dihydrogen diphosphate" "disodium dihydrogen pyrophosphate" "disodium pyrophosphate" 339ii "disodium hydrogen phosphate" "dibasic sodium phosphate" "disodium acid phosphate" "disodium hydrogen monophosphate" "disodium hydrogen phosphate" "disodium phosphate" "secondary sodium phosphate" 297 "fumaric acid" 575 "glucono delta-lactone" gdl "d-gluconic acid delta-lactone" "glucono-delta-lactone" gluconolactone "delta-gluconolactone" 270 "lactic acid, l-, d- and dl-" 504i "magnesium carbonate" 450ix "magnesium dihydrogen diphosphate" 343i "magnesium dihydrogen phosphate" "acid magnesium phosphate" "magnesium biphosphate" "magnesium dihydrogen phosphate" "magnesium phosphate mono basic" "monomagnesium orthophosphate" 580 "magnesium gluconate" 343ii "magnesium hydrogen phosphate" "dibasic magnesium phosphate" "dimagnesium phosphate" "magnesium hydrogen phosphate" "magnesium phosphate, dibasic" "secondary magnesium phosphate" 528 "magnesium hydroxide" 504ii "magnesium hydroxide carbonate" "hydrated basic magnesium carbonate" "magnesium carbonate hydroxide" "magnesium hydroxide carbonate" "magnesium subcarbonate" 329 "magnesium lactate, dl-" 530 "magnesium oxide" 296 "malic acid, dl-" "2-hydroxybutanedioic acid" "pomalous acid" 451ii "pentapotassium triphosphate" "pentapotassium tripolyphosphate" "potassium triphosphate" "potassium tripolyphosphate" 451i "pentasodium triphosphate" "pentasodium tripolyphosphate" "sodium triphosphate" "sodium tripolyphosphate" triphosphate 338 "phosphoric acid" 261i "potassium acetate" 501i "potassium carbonate" 332i "potassium dihydrogen citrate" "monopotassium citrate" "potassium citrate monobasic" 340i "potassium dihydrogen phosphate" "monobasic potassium phosphate" "monopotassium dihydrogen monophosphate" "monopotassium dihydrogen orthophosphate" "monopotassium monophosphate" "potassium acid phosphate" "potassium biphosphate" "potassium dihydrogen phosphate" 577 "potassium gluconate" 501ii "potassium hydrogen carbonate" "potassium bicarbonate" 326 "potassium lactate" 452ii "potassium lactate" "potassium metaphosphate" 337 "potassium sodium l(+)-tartrate" "potassium sodium dextro-tartrate" "rochelle salt" "seignette salt" 515i "potassium sulfate" 350ii "sodium dl-malate" "malic acid sodium salt" 335ii "sodium l(+)-tartrate" "sodium dextro-tartrate" "sodium tartrate, l (+)-" 262i "sodium acetate" 541i "sodium aluminium phosphate, acidic" salp 541ii "sodium aluminium phosphate, basic" kasal 452iii "sodium calcium polyphosphate" 500i "sodium carbonate" "soda ash" 262ii "sodium diacetate" 331i "sodium dihydrogen citrate" "monosodium citrate" "sodium citrate monobasic" 339i "sodium dihydrogen phosphate" "monobasic sodium phosphate" "monosodium dihydrogen monophosphate" "monosodium dihydrogen orthophosphate" "monosodium monophosphate" "sodium acid phosphate" "sodium biphosphate" "sodium dihydrogen phosphate" 365 "sodium fumarate" "monosodium fumarate" 350i "sodium hydrogen dl-malate" 500ii "sodium hydrogen carbonate" "baking soda" "bicarbonate of soda" "sodium bicarbonate" 514ii "sodium hydrogen sulfate" 524 "sodium hydroxide" "caustic soda" lye "sodium hydrate" 325 "sodium lactate" 452i "sodium polyphosphate" "graham's salt" "sodium hexametaphosphate" "sodium tetrapolyphosphate" 500iii "sodium sesquicarbonate" "sodium monohydrogendicarbonate" 514i "sodium sulfate" 334 "l(+)-tartaric acid" 450v "tetrapotassium diphosphate" "potassium pyrophosphate" "tetrapotassium pyrophosphate" 380 "triammonium citrate" 333iii "tricalcium citrate" 341iii "tricalcium phosphate" "calcium phosphate, tribasic" "precipitated calcium phosphate" "tricalcium phosphate" 343iii "trimagnesium phosphate" "magnesium phosphate, tribasic" "tertiary magnesium phosphate" "trimagnesium phosphate" 332ii "tripotassium citrate" "potassium citrate" 340iii "tripotassium phosphate" "potassium phosphate" "tribasic potassium phosphate" "tripotassium phosphate" 331iii "trisodium citrate" "sodium citrate" 450ii "trisodium diphosphate" "acid trisodium pyrophosphate" "trisodium monohydrogen diphosphate" 339iii "trisodium phosphate" "sodium phosphate" "tribasic sodium phosphate" "trisodium phosphate" |
| Anticaking agents | 559 "anticaking agent" "aluminium silicate" kaolin 542 "bone phosphate" 556 "calcium aluminium silicate" "aluminium calcium silicate" "calcium aluminosilicate" "calcium silicoaluminate" "sodium calcium silicoaluminate" 170i "calcium carbonate" chalk 341i "calcium dihydrogen phosphate" "acid calcium phosphate" "calcium biphosphate" "calcium dihydrogen phosphate" "calcium dihydrogen tetraoxophosphate" "monobasic calcium phosphate" "monocalcium orthophosphate" "monocalcium phosphate" 538 "calcium ferrocyanide" "hexacyanoferrate of calcium" "yellow prussiate of lime" 341ii "calcium hydrogen phosphate" "calcium hydrogen monophosphate" "calcium hydrogen orthophosphate" "calcium hydrogen phosphate" "dibasic calcium phosphate" "dicalcium phophate" "secondary calcium phosphate" 552 "calcium silicate" 903 "carnauba wax" 1503 "castor oil" "ricinus oil" 381 "ferric ammonium citrate" "ammonium ferric citrate" "ammonium iron (iii) citrate" "ammonium iron citrate" "iron ammonium citrate" 1442 "hydroxypropyl distarch phosphate" 953 isomalt "hydrogenated isomaltulose" isomaltitol 504i "magnesium carbonate" 343i "magnesium dihydrogen phosphate" "acid magnesium phosphate" "magnesium biphosphate" "magnesium dihydrogen phosphate" "magnesium phosphate mono basic" "monomagnesium orthophosphate" 343ii "magnesium hydrogen phosphate" "dibasic magnesium phosphate" "dimagnesium phosphate" "magnesium hydrogen phosphate" "magnesium phosphate, dibasic" "secondary magnesium phosphate" 504ii "magnesium hydroxide carbonate" "hydrated basic magnesium carbonate" "magnesium carbonate hydroxide" "magnesium hydroxide carbonate" "magnesium subcarbonate" 530 "magnesium oxide" 553i "magnesium silicate, synthetic" 470iii "magnesium stearate" 421 mannitol mannite "d-mannitol" 460i "microcrystalline cellulose" "cellulose gel" 900a "polydimethylsiloxane" "dimethylpolysiloxane" "dimethylsilicone fluid" "dimethylsilicone oil" "poly(dimethylsiloxane)" 536 "potassium ferrocyanide" "hexacyanoferrate of potassium" "yellow prussiate of potash" 460ii "powdered cellulose" 470i "salts of myristic, palmitic and stearic acids with ammonia, calcium, potassium and sodium" 470ii "salts of oleic acid with calcium, potassium and sodium" 551 "silicon dioxide, amorphous" silica 554 "sodium aluminium silicate" "sodium silicoaluminate" 500i "sodium carbonate" "soda ash" 535 "sodium ferrocyanide" "hexacyanoferrate of sodium" "yellow prussiate of soda" 500ii "sodium hydrogen carbonate" "baking soda" "bicarbonate of soda" "sodium bicarbonate" 500iii "sodium sesquicarbonate" "sodium monohydrogendicarbonate" 553iii talcum talc 341iii "tricalcium phosphate" "calcium phosphate, tribasic" "precipitated calcium phosphate" "tricalcium phosphate" 343iii "trimagnesium phosphate" "magnesium phosphate, tribasic" "tertiary magnesium phosphate" "trimagnesium phosphate" |
| Antioxidants | antioxidant 300 "ascorbic acid, l-" "ascorbic acid" 304 "ascorbyl palmitate" "vitamin c palmitate" 305 "ascorbyl stearate" "vitamin C stearate" 320 "butylated hydroxyanisole" bha 321 "butylated hydroxytoluene" bht 302 "calcium ascorbate" 385 "calcium disodium ethylenediaminetetraacetate" "calcium disodium edetate" "calcium disodium edta" 330 "citric acid" 472c "citric and fatty acid esters of glycerol" citrem "citric acid esters of mono- and di-glycerides" "citroglycerides" 389 "dilauryl thiodipropionate" 386 "disodium ethylenediaminetetraacetate" "calcium disodium (ethylene-dinitrilo)-tetraacetate" "calcium disodium edetate" "calcium disodium edta" "calcium disodium ethylenediaminetetraacetate" "disodium dihydrogen (ethylene-dinitrilo) - tetraacetate" "disodium edetate" "disodium edta" "disodium ethylenediaminetetraacetate" 315 "erythorbic acid" "isoascorbic acid" 1102 "glucose oxidase" "aero-glucose dehydrogenase" "glucose aerodehydrogenase" "glucose oxyhydrase" notatin 314 "guaiac resin" "guaiac gum" guaiacum "gum guaiac" 384 "isopropyl citrates" "isopropyl citrate mixture" 322i lecithin phosphatides phospholipids 322ii "lecithin, partially hydrolysed" 942 "nitrous oxide" "dinitrogen monoxide" "nitrogen oxide" 338 "phosphoric acid" 326 "potassium lactate" 224 "potassium metabisulfite" 225 "potassium sulfite" 310 "propyl gallate" 301 "sodium ascorbate" 316 "sodium erythorbate" "sodium isoascorbate" 222 "sodium hydrogen sulfite" "sodium bisulfite" 325 "sodium lactate" 223 "sodium metabisulfite" 221 "sodium sulfite" "disodium sulfite" 539 "sodium thiosulfate" "sodium hyposulfite" 512 "stannous chloride" "tin dichloride" 484 "stearyl citrate" 220 "sulfur dioxide" 334 "l(+)-tartaric acid" 319 "tertiary butylhydroquinone" tbhq 388 "thiodipropionic acid" 307b "tocopherol concentrate, mixed" "vitamin e concentrate" 307a "d-alpha-tocopherol" "(+)-alpha-tocopherol" "rrr-alpha -tocopherol" "5,7,8-trimethyltocol" 307c "dl-alpha-tocopherol" 333iii "tricalcium citrate" 332ii "tripotassium citrate" "potassium citrate" |
| Bleaching agents | 928 "bleaching agent" "benzoyl peroxide" "benzoyl superoxide" 224 "potassium metabisulfite" 223 "sodium metabisulfite" 221 "sodium sulfite" "disodium sulfite" 220 "sulfur dioxide" |
| Carriers | carrier 406 agar "agar-agar" "bengal, ceylon, chinese or japanese isinglass" "gelose" "japan agar" "layor carang" 400 "alginic acid" 403 "ammonium alginate" 901 beeswax 404 "calcium alginate" 902 "candelilla wax" 903 "carnauba wax" 407 "carrageenan" "danish agar" "eucheuman" "furcellaran agar" hypnean "irish moss gelose" iridophycan 1503 "castor oil" "ricinus oil" 459 "cyclodextrin, beta-" bcd betacd "beta-schardinger dextrin" "cyclodextrin b" 1504i cyclotetraglucose 1504ii "cyclotetraglucose syrup" 1400 "dextrins, roasted starch" 462 "ethyl cellulose" 414 "gum arabic" "acacia gum" "arabic gum" 419 "gum ghatti" 425 "konjac flour" "konjac mannen" konjac konnyaku konnyaleu 504ii "magnesium hydroxide carbonate" "hydrated basic magnesium carbonate" "magnesium carbonate hydroxide" "magnesium hydroxide carbonate" "magnesium subcarbonate" 1205 "methacrylate copolymer, basic" bmc 460i "microcrystalline cellulose" "cellulose gel" 1521 "polyethylene glycol" macrogol 402 "potassium alginate" 407a "processed eucheuma seaweed" "png-carrageenan" "semi-refined carrageenan" 1520 "propylene glycol" "methyl glycol" propanediol 405 "propylene glycol alginate" "hydroxypropyl alginate" "propane 1,2-diol alginate" ",2-propane-diol ester of alginic acid" 551 "silicon dioxide, amorphous" silica 401 "sodium alginate" 1518 triacetin 1505 "triethyl citrate" "ethyl citrate" |
| Colour retention agents | 523 "color retention agent" "colour retention agent" "aluminium ammonium sulfate" "ammonium alum" 385 "calcium disodium ethylenediaminetetraacetate" "calcium disodium edetate" "calcium disodium edta" 330 "citric acid" 386 "disodium ethylenediaminetetraacetate" "calcium disodium (ethylene-dinitrilo)-tetraacetate" "calcium disodium edetate" "calcium disodium edta" "calcium disodium ethylenediaminetetraacetate" "disodium dihydrogen (ethylene-dinitrilo) - tetraacetate" "disodium edetate" "disodium edta" "disodium ethylenediaminetetraacetate" 579 "ferrous gluconate" "iron gluconate" 585 "ferrous lactate" "iron (ii) lactate" 504i "magnesium carbonate" 511 "magnesium chloride" "magnesium chloride hexahydrate" 528 "magnesium hydroxide" 504ii "magnesium hydroxide carbonate" "hydrated basic magnesium carbonate" "magnesium carbonate hydroxide" "magnesium hydroxide carbonate" "magnesium subcarbonate" 1202 "polyvinylpyrrolidone, insoluble" 252 "potassium nitrate" nitre saltpetre 249 "potassium nitrite" 251 "sodium nitrate" "chile saltpetre" "cubic nitre" "soda nitre" 250 "sodium nitrite" 512 "stannous chloride" "tin dichloride" |
| Firming agents | 523 "aluminium ammonium sulfate" "ammonium alum" "firming agent" 520 "aluminium sulfate" 170i "calcium carbonate" chalk 509 "calcium chloride" 341i "calcium dihydrogen phosphate" "acid calcium phosphate" "calcium biphosphate" "calcium dihydrogen phosphate" "calcium dihydrogen tetraoxophosphate" "monobasic calcium phosphate" "monocalcium orthophosphate" "monocalcium phosphate" 578 "calcium gluconate" "calcium di-d-gluconate monohydrate" "calcium di-gluconate" 341ii "calcium hydrogen phosphate" "calcium hydrogen monophosphate" "calcium hydrogen orthophosphate" "calcium hydrogen phosphate" "dibasic calcium phosphate" "dicalcium phophate" "secondary calcium phosphate" 526 "calcium hydroxide" "slaked lime" 327 "calcium lactate" 516 "calcium sulfate" 424 curdlan "beta-1,3-glucan" 450vi "dicalcium diphosphate" "calcium pyrophosphate" "dicalcium pyrophosphate" 511 "magnesium chloride" "magnesium chloride hexahydrate" 580 "magnesium gluconate" 518 "magnesium sulfate" 508 "potassium chloride" sylvine sylvite 466 "sodium carboxymethyl cellulose" "cellulose gum" cmc "na cmc" "sodium cellulose glycolate" "sodium cmc" 333iii "tricalcium citrate" 341iii "tricalcium phosphate" "calcium phosphate, tribasic" "precipitated calcium phosphate" "tricalcium phosphate" |
| Flour treatment agents | 510 "flour treatment agent" "ammonium muriate" "sal ammoniac" "ammonium chloride" 342i "ammonium dihydrogen phosphate" "acid ammonium phosphate" "ammonium dihydrogen orthophosphate" "ammonium dihydrogen phosphate" "ammonium dihydrogen tetraoxophosphate" "monoammonium monophosphate" "monoammonium phosphate" "monobasic ammonium phosphate" "primary ammonium phosphate" 1100i "alpha-amylase from aspergillus oryzae var" diastase glycogenase ptyalin 1100iv "alpha-amylase from bacillus megaterium expressed in bacillus subtilis" 1100v "alpha-amylase from bacillus stearothermophilus expressed in bacillus subtilis" 1100ii "alpha-amylase from bacillus stearothermophilus" 1100iii "alpha-amylase from bacillus subtilis" glycogenase 300 "ascorbic acid" 927a azodicarbonamide azobisformamide 928 "benzoyl peroxide" "benzoyl superoxide" 1101iii bromelain 170i "calcium carbonate" chalk 341i "calcium dihydrogen phosphate" "acid calcium phosphate" "calcium biphosphate" "calcium dihydrogen phosphate" "calcium dihydrogen tetraoxophosphate" "monobasic calcium phosphate" "monocalcium orthophosphate" "monocalcium phosphate" 341ii "calcium hydrogen phosphate" "calcium hydrogen monophosphate" "calcium hydrogen orthophosphate" "calcium hydrogen phosphate" "dibasic calcium phosphate" "dicalcium phophate" "secondary calcium phosphate" 327 "calcium lactate" 529 "calcium oxide" 482i "calcium stearoyl lactylate" "calcium stearoyl-2-lactylate" "calcium stearoyl lactate" 516 "calcium sulfate" 1100vi "carbohydrase from bacillus licheniformis" 925 chlorine 472c "citric and fatty acid esters of glycerol" citrem "citric acid esters of mono- and di-glycerides" citroglycerides 342ii "diammonium hydrogen phosphate" "ammonium dihydrogen orthophosphate" "ammonium dihydrogen phosphate" "ammonium dihydrogen tetraoxophosphate" "diammonium hydrogenorthophosphate" "diammonium hydrogen phosphate" "diammonium hydrogentetraoxophosphate" "diammonium phosphate" "dibasic ammonium phosphate" 322i lecithin phosphatides phospholipids 504i "magnesium carbonate" 329 "magnesium lactate, dl" 224 "potassium metabisulfite" 1101i "protease from aspergillus orizae var" 301 "sodium ascorbate" 223 "sodium metabisulfite" 481i "sodium stearoyl lactylate" "sodium stearoyl-2-lactylate" "sodium stearoyl lactate" 221 "sodium sulfite" "disodium sulfite" 483 "stearyl tartrate" "stearyl palmityl tartrate" 220 "sulfur dioxide" 341iii "tricalcium phosphate" "calcium phosphate, tribasic" "precipitated calcium phosphate" "tricalcium phosphate" |
| Humectants | humectant 406 agar "agar-agar" "bengal, ceylon, chinese or japanese isinglass" "gelose" "japan agar" "layor carang" 400 "alginic acid" 403 "ammonium alginate" 452v "ammonium polyphosphate" 542 "bone phosphate" 404 "calcium alginate" 450vii "calcium dihydrogen diphosphate" "acid calcium pyrophosphate" "monocalcium dihydrogen pyrophosphate" 341i "calcium dihydrogen phosphate" "acid calcium phosphate" "calcium biphosphate" "calcium dihydrogen phosphate" "calcium dihydrogen tetraoxophosphate" "monobasic calcium phosphate" "monocalcium orthophosphate" "monocalcium phosphate" 341ii "calcium hydrogen phosphate" "calcium hydrogen monophosphate" "calcium hydrogen orthophosphate" "calcium hydrogen phosphate" "dibasic calcium phosphate" "dicalcium phophate" "secondary calcium phosphate" 452iv "calcium polyphosphate" 407 "carrageenan" "danish agar" "eucheuman" "furcellaran agar" hypnean "irish moss gelose" iridophycan 480 "dioctyl sodium sulfosuccinate" dss "docusate sodium" 340ii "dipotassium hydrogen phosphate" "dibasic potassium phosphate" "dipotassium acid phosphate" "dipotassium hydrogen monophosphate" "dipotassium hydrogen orthophosphate" "dipotassium hydrogen phosphate" "dipotassium monophosphate" "dipotassium phosphate" "secondary potassium phosphate" 450i "disodium diphosphate" "acid sodium pyrophosphate" "disodium dihydrogen diphosphate" "disodium dihydrogen pyrophosphate" "disodium pyrophosphate" 339ii "disodium hydrogen phosphate" "dibasic sodium phosphate" "disodium acid phosphate" "disodium hydrogen monophosphate" "disodium hydrogen phosphate" "disodium phosphate" "secondary sodium phosphate" 968 erythritol erythrite "meso-erythritol" "tetrahydroxybutane" 422 glycerol glycerin 425 "konjac flour" "konjac mannen" konjac konnyaku konnyaleu 965i maltitol "d-maltitol" "dried maltitol syrup" "hydrogenated glucose syrup" "hydrogenated high maltose-content glucose syrup" "hydrogenated maltose" "maltitol syrup powder" 965ii "maltitol syrup" 421 mannitol mannite "d-mannitol" 451ii "pentapotassium triphosphate" "pentapotassium tripolyphosphate" "potassium triphosphate" "potassium tripolyphosphate" 451i "pentasodium triphosphate" "pentasodium tripolyphosphate" "sodium triphosphate" "sodium tripolyphosphate" triphosphate 1200 polydextroses 402 "potassium alginate" 340i "potassium dihydrogen phosphate" "monobasic potassium phosphate" "monopotassium dihydrogen monophosphate" "monopotassium dihydrogen orthophosphate" "monopotassium monophosphate" "potassium acid phosphate" "potassium biphosphate" "potassium dihydrogen phosphate" 326 "potassium lactate" 452ii "potassium polyphosphate" "potassium metaphosphate" 460ii "powdered cellulose" 407a "processed eucheuma seaweed" "png-carrageenan" "semi-refined carrageenan" 1520 "propylene glycol" "methyl glycol" propanediol 350ii "sodium dl-malate" "malic acid sodium salt" 401 "sodium alginate" 452iii "sodium calcium polyphosphate" 466 "sodium carboxymethyl cellulose" "cellulose gum" cmc "na cmc" "sodium cmc" "sodium cellulose glycolate" 339i "sodium dihydrogen phosphate" "monobasic sodium phosphate" "monosodium dihydrogen monophosphate" "monosodium dihydrogen orthophosphate" "monosodium monophosphate" "sodium acid phosphate" "sodium biphosphate" "sodium dihydrogen phosphate" 350i "sodium hydrogen dl-malate" 325 "sodium lactate" 452i "sodium polyphosphate" "graham's salt" "sodium hexametaphosphate" "sodium tetrapolyphosphate" 420i sorbitol "d-glucitol" "d-glucitol syrup" sorbit "d-sorbitol" sorbol 420ii "sorbitol syrup" 450v "tetrapotassium diphosphate" "potassium pyrophosphate" "tetrapotassium pyrophosphate" 450iii "tetrasodium diphosphate" "sodium pyrophosphate" "tetrasodium pyrophosphate" 1518 triacetin 341iii "tricalcium phosphate" "calcium phosphate, tribasic" "precipitated calcium phosphate" "tricalcium phosphate" 340iii "tripotassium phosphate" "potassium phosphate" "tribasic potassium phosphate" "tripotassium phosphate" 339iii "trisodium phosphate" "sodium phosphate" "tribasic sodium phosphate" "trisodium phosphate" 450ii "trisodium diphosphate" "acid trisodium pyrophosphate" "trisodium monohydrogen diphosphate" 967 xylitol |
| Packaging gases | 290 "carbon dioxide" "carbonic acid anhydride" "dry ice" 941 nitrogen 942 "nitrous oxide" "dinitrogen monoxide" "nitrogen oxide" |
| Preservatives | preservative 260 "acetic acid" 210 "benzoic acid" 928 "benzoyl peroxide" "benzoyl superoxide" 263 "calcium acetate" 213 "calcium benzoate" "monocalcium benzoate" 385 "calcium disodium ethylenediaminetetraacetate" "calcium disodium edetate" "calcium disodium edta" 282 "calcium propionate" "calcium propanoate" 203 "calcium sorbate" 290 "carbon dioxide" "dry ice" "carbonic acid anhydride" 242 "dimethyl dicarbonate" dmdc "dimethyl pyrocarbonate" 230 diphenyl 386 "disodium ethylenediaminetetraacetate" "calcium disodium (ethylene-dinitrilo)-tetraacetate" "calcium disodium edetate" "calcium disodium edta" "calcium disodium ethylenediaminetetraacetate" "disodium dihydrogen (ethylene-dinitrilo) - tetraacetate" "disodium edetate" "disodium edta" "disodium ethylenediaminetetraacetate" 214 "ethyl para-hydroxybenzoate" "ethyl p-oxybenzoate" ethylparaben 239 "hexamethylene tetramine" hexamine methenamine 384 "isopropyl citrates" "isopropyl citrate mixture" 243 "lauric arginate ethyl ester" 1105 lysozyme 218 "methyl para-hydroxybenzoate" "methylparaben" "methyl p-oxybenzoate" 235 natamycin pimaricin 234 nisin 231 "ortho-phenylphenol" orthoxenol 261i "potassium acetate" 212 "potassium benzoate" 224 "potassium metabisulfite" 252 "potassium nitrate" nitre saltpetre 249 "potassium nitrite" 283 "potassium propionate" 202 "potassium sorbate" 225 "potassium sulfite" 280 "propionic acid" 262i "sodium acetate" 211 "sodium benzoate" 262ii "sodium diacetate" 222 "sodium hydrogen sulfite" "sodium bisulfite" 223 "sodium metabisulfite" 251 "sodium nitrate" "chile saltpetre" "cubic nitre" "soda nitre" 250 "sodium nitrite" 232 "sodium ortho-phenylphenol" 281 "sodium propionate" 221 "sodium sulfite" "disodium sulfite" 200 "sorbic acid" 220 "sulfur dioxide" 339iii "trisodium phosphate" "sodium phosphate" "tribasic sodium phosphate" "trisodium phosphate" |
| Propellants | propellant 290 "carbon dioxide" "carbonic acid anhydride" "dry ice" 941 nitrogen 942 "nitrous oxide" "dinitrogen monoxide" "nitrogen oxide" |
| Raising agents | 523 "aluminium ammonium sulfate" "ammonium alum" "raising agent" 503i "ammonium carbonate" 342i "ammonium dihydrogen phosphate" "acid ammonium phosphate" "ammonium dihydrogen orthophosphate" "ammonium dihydrogen phosphate" "ammonium dihydrogen tetraoxophosphate" "monoammonium monophosphate" "monoammonium phosphate" "monobasic ammonium phosphate" "primary ammonium phosphate" 503ii "ammonium hydrogen carbonate" "ammonium bicarbonate" 450vii "calcium dihydrogen diphosphate" "acid calcium pyrophosphate" "monocalcium dihydrogen pyrophosphate" 341i "calcium dihydrogen phosphate" "acid calcium phosphate" "calcium biphosphate" "calcium dihydrogen phosphate" "calcium dihydrogen tetraoxophosphate" "monobasic calcium phosphate" "monocalcium orthophosphate" "monocalcium phosphate" 341ii "calcium hydrogen phosphate" "calcium hydrogen monophosphate" "calcium hydrogen orthophosphate" "calcium hydrogen phosphate" "dibasic calcium phosphate" "dicalcium phophate" "secondary calcium phosphate" 452iv "calcium polyphosphate" 342ii "diammonium hydrogen phosphate" "ammonium dihydrogen orthophosphate" "ammonium dihydrogen phosphate" "ammonium dihydrogen tetraoxophosphate" "diammonium hydrogenorthophosphate" "diammonium hydrogen phosphate" "diammonium hydrogentetraoxophosphate" "diammonium phosphate" "dibasic ammonium phosphate" 450vi "dicalcium diphosphate" "calcium pyrophosphate" "dicalcium pyrophosphate" 450i "disodium diphosphate" "acid sodium pyrophosphate" "disodium dihydrogen diphosphate" "disodium dihydrogen pyrophosphate" "disodium pyrophosphate" 575 "glucono delta-lactone" gdl "d-gluconic acid delta-lactone" "glucono-delta-lactone" gluconolactone "delta-gluconolactone" 450ix "magnesium dihydrogen diphosphate" 343ii "magnesium hydrogen phosphate" "dibasic magnesium phosphate" "dimagnesium phosphate" "magnesium hydrogen phosphate" "magnesium phosphate, dibasic" "secondary magnesium phosphate" 501ii "potassium hydrogen carbonate" "potassium bicarbonate" 452ii "potassium polyphosphate" "potassium metaphosphate" 541i "sodium aluminium phosphate, acidic" salp 452iii "sodium calcium polyphosphate" 500i "sodium carbonate" "soda ash" 339i "sodium dihydrogen phosphate" "monobasic sodium phosphate" "monosodium dihydrogen monophosphate" "monosodium dihydrogen orthophosphate" "monosodium monophosphate" "sodium acid phosphate" "sodium biphosphate" "sodium dihydrogen phosphate" 500ii "sodium hydrogen carbonate" "baking soda" "bicarbonate of soda" "sodium bicarbonate" 452i "sodium polyphosphate" "graham's salt" "sodium hexametaphosphate" "sodium tetrapolyphosphate" 500iii "sodium sesquicarbonate" "sodium monohydrogendicarbonate" 450v "tetrapotassium diphosphate" "potassium pyrophosphate" "tetrapotassium pyrophosphate" 450iii "tetrasodium diphosphate" "sodium pyrophosphate" "tetrasodium pyrophosphate" 341iii "tricalcium phosphate" "calcium phosphate, tribasic" "precipitated calcium phosphate" "tricalcium phosphate" 450ii "trisodium diphosphate" "acid trisodium pyrophosphate" "trisodium monohydrogen diphosphate" |
| Sequestrants | sequestrant 472a "acetic and fatty acid esters of glycerol" "acetic acid esters of mono- and diglycerides" acetoglycerides "acetylated mono- and diglycerides" 400 "alginic acid" 403 "ammonium alginate" 452v "ammonium polyphosphate" 300 "ascorbic acid" 404 "calcium alginate" 450vii "calcium dihydrogen diphosphate" "acid calcium pyrophosphate" "monocalcium dihydrogen pyrophosphate" 341i "calcium dihydrogen phosphate" "acid calcium phosphate" "calcium biphosphate" "calcium dihydrogen phosphate" "calcium dihydrogen tetraoxophosphate" "monobasic calcium phosphate" "monocalcium orthophosphate" "monocalcium phosphate" 385 "calcium disodium ethylenediaminetetraacetate" "calcium disodium edetate" "calcium disodium edta" 578 "calcium gluconate" "calcium di-d-gluconate monohydrate" "calcium di-gluconate" 452iv "calcium polyphosphate" 516 "calcium sulfate" 330 "citric acid" 472c "citric and fatty acid esters of glycerol" citrem "citric acid esters of mono- and di-glycerides" citroglycerides 472e "diacetyltartaric and fatty acid esters of glycerol" datem "diacetyltartaric acid esters of mono- and diglycerides" "mixed acetic and tartaric acid esters of mono and diglycerides of fatty acids" "mixed glycerol esters of diacetyltartaric acid and fatty acids from food fats" "tartaric, acetic and fatty acid esters of glycerol, mixed" 450vi "dicalcium diphosphate" "calcium pyrophosphate" "dicalcium pyrophosphate" 340ii "dipotassium hydrogen phosphate" "dibasic potassium phosphate" "dipotassium acid phosphate" "dipotassium hydrogen monophosphate" "dipotassium hydrogen orthophosphate" "dipotassium hydrogen phosphate" "dipotassium monophosphate" "dipotassium phosphate" "secondary potassium phosphate" 450i "disodium diphosphate" "acid sodium pyrophosphate" "disodium dihydrogen diphosphate" "disodium dihydrogen pyrophosphate" "disodium pyrophosphate" 386 "disodium ethylenediaminetetraacetate" "calcium disodium (ethylene-dinitrilo)-tetraacetate" "calcium disodium edetate" "calcium disodium edta" "calcium disodium ethylenediaminetetraacetate" "disodium dihydrogen (ethylene-dinitrilo) - tetraacetate" "disodium edetate" "disodium edta" "disodium ethylenediaminetetraacetate" 339ii "disodium hydrogen phosphate" "dibasic sodium phosphate" "disodium acid phosphate" "disodium hydrogen monophosphate" "disodium hydrogen phosphate" "disodium phosphate" "secondary sodium phosphate" 575 "glucono delta-lactone" gdl "d-gluconic acid delta-lactone" "glucono-delta-lactone" gluconolactone "delta-gluconolactone" 384 "isopropyl citrates" "isopropyl citrate mixture" 472b "lactic and fatty acid esters of glycerol" "lactic acid esters of mono- and diglycerides" lactoglycerides 296 "malic acid, dl-" "2-hydroxybutanedioic acid" "pomalous acid" 451ii "pentapotassium triphosphate" "pentapotassium tripolyphosphate" "potassium triphosphate" "potassium tripolyphosphate" 451i "pentasodium triphosphate" "pentasodium tripolyphosphate" "sodium triphosphate" "sodium tripolyphosphate" triphosphate 338 "phosphoric acid" 402 "potassium alginate" 332i "potassium dihydrogen citrate" "monopotassium citrate" "potassium citrate monobasic" 340i "potassium dihydrogen phosphate" "monobasic potassium phosphate" "monopotassium dihydrogen monophosphate" "monopotassium dihydrogen orthophosphate" "monopotassium monophosphate" "potassium acid phosphate" "potassium biphosphate" "potassium dihydrogen phosphate" 577 "potassium gluconate" 452ii "potassium polyphosphate" "potassium metaphosphate" 337 "potassium sodium l(+)-tartrate" "potassium sodium dextro-tartrate" "rochelle salt" "seignette salt" 335ii "sodium l(+)-tartrate" "sodium dextro-tartrate" "sodium tartrate, l (+)-" 262i "sodium acetate" 401 "sodium alginate" 452iii "sodium calcium polyphosphate" 262ii "sodium diacetate" 331i "sodium dihydrogen citrate" "monosodium citrate" "sodium citrate monobasic" 339i "sodium dihydrogen phosphate" "monobasic sodium phosphate" "monosodium dihydrogen monophosphate" "monosodium dihydrogen orthophosphate" "monosodium monophosphate" "sodium acid phosphate" "sodium biphosphate" "sodium dihydrogen phosphate" 576 "sodium gluconate" "sodium d-gluconate" 452i "sodium polyphosphate" "graham's salt" "sodium hexametaphosphate" "sodium tetrapolyphosphate" 539 "sodium thiosulfate" "sodium hyposulfite" 420i sorbitol "d-glucitol" "d-glucitol syrup" sorbit "d-sorbitol" sorbol 420ii "sorbitol syrup" 484 "stearyl citrate" 334 "l(+)-tartaric acid" 450v "tetrapotassium diphosphate" "potassium pyrophosphate" "tetrapotassium pyrophosphate" 450iii "tetrasodium diphosphate" "sodium pyrophosphate" "tetrasodium pyrophosphate" 333iii "tricalcium citrate" 1505 "triethyl citrate" "ethyl citrate" 332ii "tripotassium citrate" "potassium citrate" 340iii "tripotassium phosphate" "potassium phosphate" "tribasic potassium phosphate" "tripotassium phosphate" 331iii "trisodium citrate" "sodium citrate" 450ii "trisodium diphosphate" "acid trisodium pyrophosphate" "trisodium monohydrogen diphosphate" 339iii "trisodium phosphate" "sodium phosphate" "tribasic sodium phosphate" "trisodium phosphate" |
| Stabilizers | stabilizer 472a "acetic and fatty acid esters of glycerol" "acetic acid esters of mono- and diglycerides" acetoglycerides "acetylated mono- and diglycerides" 1422 "acetylated distarch adipate" 1414 "acetylated distarch phosphate" 1451 "acetylated oxidized starch" 1401 "acid-treated starch" "starch, acid-treated" 406 agar "agar-agar" "bengal, ceylon, chinese or japanese isinglass" "gelose" "japan agar" "layor carang" 400 "alginic acid" 1402 "alkaline treated starch" "starch, alkaline treated" 523 "aluminium ammonium sulfate" "ammonium alum" 403 "ammonium alginate" 342i "ammonium dihydrogen phosphate" "acid ammonium phosphate" "ammonium dihydrogen orthophosphate" "ammonium dihydrogen phosphate" "ammonium dihydrogen tetraoxophosphate" "monoammonium monophosphate" "monoammonium phosphate" "monobasic ammonium phosphate" "primary ammonium phosphate" 452v "ammonium polyphosphate" 901 "beeswax" 1403 "bleached starch" "starch, bleached" 1101iii "bromelain" 263 "calcium acetate" 404 "calcium alginate" 170i "calcium carbonate" chalk 509 "calcium chloride" 450vii "calcium dihydrogen diphosphate" "acid calcium pyrophosphate" "monocalcium dihydrogen pyrophosphate" 341i "calcium dihydrogen phosphate" "acid calcium phosphate" "calcium biphosphate" "calcium dihydrogen phosphate" "calcium dihydrogen tetraoxophosphate" "monobasic calcium phosphate" "monocalcium orthophosphate" "monocalcium phosphate" 341ii "calcium hydrogen phosphate" "calcium hydrogen monophosphate" "calcium hydrogen orthophosphate" "calcium hydrogen phosphate" "dibasic calcium phosphate" "dicalcium phophate" "secondary calcium phosphate" 452iv "calcium polyphosphate" 482i "calcium stearoyl lactylate" "calcium stearoyl-2-lactylate" "calcium stearoyl lactate" 516 "calcium sulfate" 410 "carob bean gum" algaroba "carob gum" "locust bean gum" 407 "carrageenan" "danish agar" "eucheuman" "furcellaran agar" hypnean "irish moss gelose" iridophycan 427 "cassia gum" 472c "citric and fatty acid esters of glycerol" citrem "citric acid esters of mono- and di-glycerides" citroglycerides 468 "cross-linked sodium carboxymethyl cellulose" "cross-linked-cellulose gum" 424 curdlan "beta-1,3-glucan" 457 "cyclodextrin, alpha" 459 "cyclodextrin, beta" bcd betacd "cyclodextrin b" "beta-schardinger dextrin" 458 "cyclodextrin, gamma" 1400 "dextrins, roasted starch" 472e "diacetyltartaric and fatty acid esters of glycerol" datem "diacetyltartaric acid esters of mono- and diglycerides" "mixed acetic and tartaric acid esters of mono and diglycerides of fatty acids" "mixed glycerol esters of diacetyltartaric acid and fatty acids from food fats" "tartaric, acetic and fatty acid esters of glycerol, mixed" 342ii "diammonium hydrogen phosphate" "ammonium dihydrogen orthophosphate" "ammonium dihydrogen phosphate" "ammonium dihydrogen tetraoxophosphate" "diammonium hydrogenorthophosphate" "diammonium hydrogen phosphate" "diammonium hydrogentetraoxophosphate" "diammonium phosphate" "dibasic ammonium phosphate" 450vi "dicalcium diphosphate" "calcium pyrophosphate" "dicalcium pyrophosphate" 450i "disodium diphosphate" "acid sodium pyrophosphate" "disodium dihydrogen diphosphate" "disodium dihydrogen pyrophosphate" "disodium pyrophosphate" 386 "disodium ethylenediaminetetraacetate" "calcium disodium (ethylene-dinitrilo)-tetraacetate" "calcium disodium edetate" "calcium disodium edta" "calcium disodium ethylenediaminetetraacetate" "disodium dihydrogen (ethylene-dinitrilo) - tetraacetate" "disodium edetate" "disodium edta" "disodium ethylenediaminetetraacetate" 339ii "disodium hydrogen phosphate" "dibasic sodium phosphate" "disodium acid phosphate" "disodium hydrogen monophosphate" "disodium hydrogen phosphate" "disodium phosphate" "secondary sodium phosphate" 1412 "distarch phosphate" 467 "ethyl hydroxyethyl cellulose" 418 "gellan gum" 445iii "glycerol ester of wood rosin" "ester gum" 412 "guar gum" "guar flour" "gum cyamopsis" 414 "gum arabic" "acacia gum" "arabic gum" 419 "gum ghatti" 463 "hydroxypropyl cellulose" "cellulose hydroxypropyl ether" "modified cellulose" 1442 "hydroxypropyl distarch phosphate" 464 "hydroxypropyl methyl cellulose" 1440 "hydroxypropyl starch" 1103 "invertases" 953 isomalt "hydrogenated isomaltulose" isomaltitol 416 "karaya gum" "gum karaya" "gum sterculia" "kadaya" "karaya" "katilo" "kullo" "kutterra" "sterculia" 425 "konjac flour" "konjac mannen" konjac konnyaku konnyaleu 472b "lactic and fatty acid esters of glycerol" "lactic acid esters of mono- and diglycerides" lactoglycerides 511 "magnesium chloride" "magnesium chloride hexahydrate" 450ix "magnesium dihydrogen diphosphate" 343i "magnesium dihydrogen phosphate" "acid magnesium phosphate" "magnesium biphosphate" "magnesium dihydrogen phosphate" "magnesium phosphate mono basic" "monomagnesium orthophosphate" 343ii "magnesium hydrogen phosphate" "dibasic magnesium phosphate" "dimagnesium phosphate" "magnesium hydrogen phosphate" "magnesium phosphate, dibasic" "secondary magnesium phosphate" 965i maltitol "d-maltitol" "dried maltitol syrup" "hydrogenated glucose syrup" "hydrogenated high maltose-content glucose syrup" "hydrogenated maltose" "maltitol syrup powder" 965ii "maltitol syrup" 421 mannitol mannite "d-mannitol" 461 "methyl cellulose" "cellulose methyl ether" "methyl ether of cellulose" 465 "methyl ethyl cellulose" "methyl ethyl ether of cellulose" 460i "microcrystalline cellulose" "cellulose gel" 471 "mono- and di-glycerides of fatty acids" "glyceryl monooleate" "glyceryl monoplamitate" "glyceryl monostearate" gms monoolein monopalmitin monostearin 1410 "monostarch phosphate" 1404 "oxidized starch" 440 pectin 451ii "pentapotassium triphosphate" "pentapotassium tripolyphosphate" "potassium triphosphate" "potassium tripolyphosphate" 451i "pentasodium triphosphate" "pentasodium tripolyphosphate" "sodium triphosphate" "sodium tripolyphosphate" triphosphate 1413 "phosphated distarch phosphate" 1200 polydextroses 475 "polyglycerol esters of fatty acids" "glycerin fatty acid esters" "polyglycerol fatty acid esters" 432 "polyoxyethylene (20) sorbitan monolaurate" "polysorbate 20" 433 "polyoxyethylene (20) sorbitan monooleate" "polysorbate 80" 435 "polyoxyethylene (20) sorbitan monostearate" "polysorbate 60" 436 "polyoxyethylene (20) sorbitan tristearate" "polysorbate 65" 1209 "polyvinyl alcohol (pva)" pva "polyethylene glycol graft copolymer" 1201 polyvinylpyrrolidone povidone pvp 1202 402 "potassium alginate" 501i "potassium carbonate" 508 "potassium chloride" sylvine sylvite 332i "potassium dihydrogen citrate" "monopotassium citrate" "potassium citrate monobasic" 340i "potassium dihydrogen phosphate" "monobasic potassium phosphate" "monopotassium dihydrogen monophosphate" "monopotassium dihydrogen orthophosphate" "monopotassium monophosphate" "potassium acid phosphate" "potassium biphosphate" "potassium dihydrogen phosphate" 501ii "potassium hydrogen carbonate" "potassium bicarbonate" 452ii "potassium polyphosphate" "potassium metaphosphate" 337 "potassium sodium l(+)-tartrate" "potassium sodium dextro-tartrate" "rochelle salt" "seignette salt" 460ii "powdered cellulose" 407a "processed eucheuma seaweed" "png-carrageenan" "semi-refined carrageenan" 405 "propylene glycol alginate" "hydroxypropyl alginate" "propane 1,2-diol alginate" ",2-propane-diol ester of alginic acid" 1101i "protease from aspergillus orizae var" 470i "salts of myristic, palmitic and stearic acids with ammonia, calcium, potassium and sodium" 470ii "salts of oleic acid with calcium, potassium and sodium" 335ii "sodium l(+)-tartrate" "sodium dextro-tartrate" "sodium tartrate, l (+)-" 401 "sodium alginate" 541i "sodium aluminium phosphate, acidic" salp 541ii "sodium aluminium phosphate, basic" kasal 452iii "sodium calcium polyphosphate" 500i "sodium carbonate" "soda ash" 466 "sodium carboxymethyl cellulose" "cellulose gum" cmc "na cmc" "sodium cmc" "sodium cellulose glycolate" 469 "sodium carboxymethyl cellulose, enzymatically hydrolysed" "cellulose gum, enzymatically hydrolyzed" 331i "sodium dihydrogen citrate" "monosodium citrate" "sodium citrate monobasic" 339i "sodium dihydrogen phosphate" "monobasic sodium phosphate" "monosodium dihydrogen monophosphate" "monosodium dihydrogen orthophosphate" "monosodium monophosphate" "sodium acid phosphate" "sodium biphosphate" "sodium dihydrogen phosphate" 576 "sodium gluconate" "sodium d-gluconate" 500ii "sodium hydrogen carbonate" "baking soda" "bicarbonate of soda" "sodium bicarbonate" 452i "sodium polyphosphate" "graham's salt" "sodium hexametaphosphate" "sodium tetrapolyphosphate" 481i "sodium stearoyl lactylate" "sodium stearoyl-2-lactylate" "sodium stearoyl lactate" 493 "sorbitan monolaurate" "sorbitan laurate" 494 "sorbitan monooleate" 491 "sorbitan monostearate" 492 "sorbitan tristearate" 420i sorbitol "d-glucitol" "d-glucitol syrup" sorbit "d-sorbitol" sorbol 420ii "sorbitol syrup" 1420 "starch acetate" "starch acetate esterified with acetic anhydride" "starch acetate esterified with vinyl acetate" 1450 "starch sodium octenyl succinate" 1405 "starches, enzyme treated" "starch, enzyme treated" 473a "sucrose oligoesters, type i and type ii" 444 "sucrose acetate isobutyrate" saib 473 "sucrose esters of fatty acids" "sucrose fatty acid esters" 437 "tamarind seed polysaccharide" 181 "tannic acid" tannins "gallotannic acid" 417 "tara gum" "peruvian carob" 450v "tetrapotassium diphosphate" "potassium pyrophosphate" "tetrapotassium pyrophosphate" 450iii "tetrasodium diphosphate" "sodium pyrophosphate" "tetrasodium pyrophosphate" 413 "tragacanth gum" 333iii "tricalcium citrate" 341iii "tricalcium phosphate" "calcium phosphate, tribasic" "precipitated calcium phosphate" "tricalcium phosphate" 1505 "triethyl citrate" "ethyl citrate" 343iii "trimagnesium phosphate" "magnesium phosphate, tribasic" "tertiary magnesium phosphate" "trimagnesium phosphate" 332ii "tripotassium citrate" "potassium citrate" 340iii "tripotassium phosphate" "potassium phosphate" "tribasic potassium phosphate" "tripotassium phosphate" 331iii "trisodium citrate" "sodium citrate" 450ii "trisodium diphosphate" "acid trisodium pyrophosphate" "trisodium monohydrogen diphosphate" 339iii "trisodium phosphate" "sodium phosphate" "tribasic sodium phosphate" "trisodium phosphate" 415 "xanthan gum" 967 xylitol 340ii "dipotassium hydrogen phosphate" "dibasic potassium phosphate" "dipotassium acid phosphate" "dipotassium hydrogen monophosphate" "dipotassium hydrogen orthophosphate" "dipotassium hydrogen phosphate" "dipotassium monophosphate" "dipotassium phosphate" "secondary potassium phosphate" |
| Inorganic phosphates | 101 322 338 339 340 341 342 343 442 450 451 452 541 542 627 631 635 1410 1412 1412 1414 1442 "ammonium phosphate" "calcium hexametaphosphate" "calcium hypophosphite" "calcium phosphate" "calcium pyrophosphate" "dibasic magnesium phosphate" "ferric phosphate" "ferric pyrophosphate" "ferric sodium pyrophosphate" "manganous hypophosphite" "phosphoric acid" "potassium hypophosphite" "potassium phosphate" "potassium poly-metaphosphate" "potassium polyphosphate" "potassium pyrophosphate" "potassium tripolyphosphate" "sodium acid pyrophosphate" "sodium aluminum phosphate" "sodium ferri-citro-pyrophosphate" "sodium hexametaphosphate" "sodium hypophosphite" "sodium metaphosphate" "sodium phosphate" "sodium pyrophosphate" "sodium tetra-phosphate" "sodium tetra-metaphosphate" "sodium tri-metaphosphate" "sodium tri-polyphosphate" "tribasic magnesium phosphate" |
| Nitrates | 251 "sodium nitrate" "sodium nitrite" "potassium nitrate" "potassium nitrite" |

| Supplemental Table 2. Codex Alimentarius permitted additives | | | | |
| --- | --- | --- | --- | --- |
| INS no. | PERMITTED ADDITIVES | Standard for processed cereal-based foods for infants and young children - Codex Stan 74-1981 | Standard for Canned Baby Foods - Codex Stan 73-1981 | Advisory lists of nutrient compounds for use in foods for special dietary uses intended for infants and young children - CAC/GL 10-1979 |
| **Emulsifiers** | | | | |
| 322 | Lecithins | ✓ | ✓ |  |
| 471 | Mono- and diglycerides | ✓ | ✓ |  |
| 472a | Acetic and fatty acid esters of glycerol | ✓ |  |  |
| 472b | Lactic and fatty acid esters of glycerol | ✓ |  |  |
| 472c | Citric and fatty acid esters of glycerol | ✓ |  |  |
| **Acidity Regulators** | | | | |
| 500 | Sodium carbonate | . | ✓ |  |
| 500 ii | Sodium hydrogen carbonate | ✓ | ✓ |  |
| 501 ii | Potassium hydrogen carbonate | ✓ | ✓ |  |
| 170 i | Calcium carbonate | ✓ | ✓ |  |
| 270 | L(+) Lactic acid | ✓ | ✓ |  |
| 330 | Citric acid | ✓ | ✓ |  |
| 260 | Acetic acid | ✓ | ✓ |  |
| 261 | Potassium acetates | ✓ |  |  |
| 262 i | Sodium acetate | ✓ |  |  |
| 263 | Calcium acetate | ✓ |  |  |
| 296 | Malic acid (DL) – L(+)-form only | ✓ |  |  |
| 325 | Sodium lactate (solution) – L(+)-form only | ✓ |  |  |
| 326 | Potassium lactate (solution) – L(+)- form only | ✓ |  |  |
| 327 | Calcium lactate – L(+)-form only | ✓ |  |  |
| 331 i | Monosodium citrate | ✓ |  |  |
| 331 ii | Trisodium citrate | ✓ |  |  |
| 332 i | Monopotassium citrate | ✓ |  |  |
| 332 ii | Tripotassium citrate | ✓ |  |  |
| 333 | Calcium citrate | ✓ |  |  |
| 507 | Hydrochloric acid | ✓ |  |  |
| 524 | Sodium hydroxide | ✓ |  |  |
| 525 | Potassium hydroxide | ✓ |  |  |
| 526 | Calcium hydroxide | ✓ |  |  |
| 575 | Glucono delta-lactone | ✓ |  |  |
| 334 | L(+)-Tartaric acid – L(+)form only | ✓ |  |  |
| 335 ii | Disodium tartrate | ✓ |  |  |
| 337 | Potassium sodium L(+)tartrate L(+)form only | ✓ |  |  |
| 338 | Orthophosphoric acid | ✓ |  |  |
| 339 i | Monosodium orthophosphate | ✓ |  |  |
| 339 ii | Disodium orthophosphate | ✓ |  |  |
| 339 iii | Trisodium orthophosphate | ✓ |  |  |
| 340 i | Monopotassium orthophosphate | ✓ |  |  |
| 340 ii | Dipotassium orthophosphate | ✓ |  |  |
| 340 iii | Tripotassium orthophosphate | ✓ |  |  |
| 341 i | Monocalcium orthophosphate | ✓ |  |  |
| 341 ii | Dicalcium orthophosphate | ✓ |  |  |
| 341 iii | Tricalcium orthophosphate | ✓ |  |  |
| **Antioxidants** | | | | |
| 307 | Alpha-tocopherol | ✓ | ✓ |  |
| 304 | L-Ascorbyl palmitate | ✓ | ✓ |  |
| 300 | L-Ascorbic acid | ✓ | ✓ |  |
| 301 | Sodium ascorbate | ✓ | ✓ | ✓ |
| 303 | Potassium ascorbate | ✓ | ✓ |  |
| 302 | Calcium ascorbate | ✓ | ✓ |  |
| **Raising Agents** | | | | |
| 503 i | Ammonium carbonate | ✓ |  |  |
| 503 ii | Ammonium hydrogen carbonate | ✓ |  |  |
| 500 i | Sodium carbonate | ✓ |  |  |
| 500 ii | Sodium hydrogen carbonate | ✓ |  |  |
| **Thickeners** | | | | |
| 410 | Carob bean gum | ✓ | ✓ |  |
| 412 | Guar gum | ✓ | ✓ |  |
| 414 | Gum arabic | ✓ |  | ✓ |
| 415 | Xanthan gum | ✓ |  |  |
| 440 | Pectins (Amidated and NonAmidated) | ✓ | ✓ |  |
| 421 | Mannitol |  |  | ✓ |
| 1404 | Oxidized starch | ✓ |  |  |
| 1410 | Monostarch phosphate | ✓ |  |  |
| 1412 | Distarch phosphate | ✓ | ✓ |  |
| 1413 | Phosphated distarch phosphate | ✓ | ✓ |  |
| 1414 | Acetylated distarch phosphate | ✓ | ✓ |  |
| 1422 | Acetylated distarch adipate | ✓ | ✓ |  |
| 1420 | Starch acetate esterified with acetic anhydride | ✓ |  |  |
| 1450 | Starch sodium octenyl succinate | ✓ |  | ✓ |
| 1451 | Acetylated oxidized starch | ✓ |  |  |
| 1421 | Acetylated distarch glycerol |  | ✓ |  |
| 1423 | Distarch glycerol |  | ✓ |  |
| 1440 | Hydroxypropyl starch |  | ✓ |  |
| **Anticaking Agents** | | | | |
| 551 | Silicon dioxide (amorphous) | ✓ |  | ✓ |
| **Packaging Gases** | | | | |
| 290 | Carbon dioxide | ✓ |  |  |
| 941 | Nitrogen | ✓ |  |  |

| Supplemental Table 3. Level of processing among commercially produced complementary foods, by country | | | | | | | |
| --- | --- | --- | --- | --- | --- | --- | --- |
|  | Cambodia  (n=227) | Indonesia  (n=272) | Laos PDR  (n=118) | Malaysia  (n=388) | Philippines  (n=182) | Thailand  (n=206) | Viet Nam  (n=242) |
| Group 1. Unprocessed/minimally processed | 17.2 (39) | 18.8 (51) | 31.4 (37) | 38.1 (148) | 18.1 (33) | 43.2 (89) | 18.2 (44) |
| Group. 2. Culinary ingredients | 0.0 (0) | 0.7 (2) | 0.9 (1) | 1.0 (4) | 0.0 (0) | 0.0 (0) | 3.3 (8) |
| Group 3. Processed foods | 26.0 (59) | 23.5 (64) | 44.1 (52) | 17.8 (69) | 21.4 (39) | 37.9 (78) | 15.3 (37) |
| Group 4. Ultra-processed foods | 56.8 (129) | 57.0 (155) | 23.7 (28) | 43.0 (167) | 60.4 (110) | 18.9 (39) | 63.2 (153) |
| ^1^ Values presented in %(n); PDR = People’s Democratic Republic | | | | | | | |

| Supplemental Table 4. Presence of additives in commercially produced complementary foods, by country^1^ | | | | | | |  | |  |
| --- | --- | --- | --- | --- | --- | --- | --- | --- | --- |
|  | Cambodia  (n=227) | Indonesia  (n=272) | Laos PDR  (n=118) | Malaysia  (n=388) | Philippines  (n=182) | Thailand  (n=206) | | Viet Nam  (n=242) | |
| Cosmetic additives |  |  |  |  |  |  | |  | |
| Antifoaming agent | 1. (9) | 0.0 (0) | 2.5 (3) | 0.8 (3) | 0.6 (1) | 1.0 (2) | | 0.4 (1) | |
| Bulking agent | 0.9 (2) | 0.4 (1) | 0.0 (0) | 0.5 (2) | 0.0 (0) | 1.9 (4) | | 0.0 (0) | |
| Carbonating agent | 1. (0) | 0.0 (0) | 0.0 (0) | 1. (0) | 0.0 (0) | 0.0 (0) | | 0.0 (0) | |
| Colour | 22.9 (52) | 12.9 (35) | 8.5 (10) | 21.1 (82) | 20.9 (38) | 5.8 (12) | | 31.0 (75) | |
| Emulsifier | 24.2 (55) | 32.0 (87) | 14.4 (17) | 17.0 (66) | 19.8 (36) | 7.8 (16) | | 17.4 (42) | |
| Emulsifying salt | 7.9 (18) | 1.8 (5) | 5.1 (6) | 3.1 (12) | 2.8 (5) | 2.4 (5) | | 7.4 (18) | |
| Flavour enhancer | 5.3 (12) | 0.0 (0) | 0.0 (0) | 1.6 (6) | 3.3 (6) | 1.5 (3) | | 5.4 (13) | |
| Foaming agent | - 1. (1) | 0.0 (0) | 0.0 (0) | 0.3 (1) | 1.1 (2) | 1.5 (3) | | 0.0 (0) | |
| Gelling agent | 4.0 (9) | 0.7 (2) | 2.5 (3) | 1.8 (7) | 2.8 (5) | 1.9 (4) | | 1.2 (3) | |
| Glazing agent | 5.7 (13) | 0.7 (2) | 5.1 (6) | 2.8 (11) | 3.3 (6) | 2.9 (6) | | 1.2 (3) | |
| Sweetener | 1. (0) | 0.0 (0) | 0.0 (0) | 0.0 (0) | 0.0 (0) | 1.0 (2) | | 0.0 (0) | |
| Thickener | 17.2 (39) | 17.7 (48) | 10.2 (12) | 12.4 (48) | 23.6 (43) | 7.8 (16) | | 12.8 (31) | |
| Other additives |  |  |  |  |  |  | |  | |
| Acidity regulator | 33.9 (77) | 23.9 (65) | 18.6 (22) | 27.6 (107) | 35.2 (64) | 14.1 (29) | | 45.9 (111) | |
| Anticaking agent | 15.4 (35) | 15.1 (41) | 8.5 (10) | 21.1 (82) | 20.9 (38) | 5.3 (11) | | 33.9 (82) | |
| Antioxidant | 35.2 (80) | 52.2 (142) | 17.8 (21) | 23.5 (91) | 50.6 (92) | 13.6 (28) | | 28.1 (68) | |
| Bleaching agent | 1. (0) | 0.4 (1) | 1. (0) | 1. (0) | 0.0 (0) | 0.0 (0) | | 0.0 (0) | |
| Carrier | 1.8 (4) | 0.4 (1) | 9.3 (11) | 0.5 (2) | 0.0 (0) | 6.3 (13) | | 0.4 (1) | |
| Colour retention agent | 15.4 (35) | 4.0 (11) | 10.2 (12) | 6.4 (25) | 15.4 (28) | 4.4 (9) | | 11.2 (27) | |
| Firming agent | 18.1 (41) | 9.6 (26) | 5.9 (7) | 18.6 (72) | 18.7 (34) | 2.9 (6) | | 31.4 (76) | |
| Flour treatment agent | 36.1 (82) | 42.3 (115) | 19.5 (23) | 29.6 (115) | 49.5 (90) | 13.1 (27) | | 39.7 (96) | |
| Humectant | 9.3 (21) | 1.5 (4) | 4.2 (5) | 5.2 (20) | 6.0 (11) | 2.9 (6) | | 6.6 (16) | |
| Packaging gas | 1. (0) | 0.0 (0) | 0.0 (0) | 0.0 (0) | 0.0 (0) | 1.0 (2) | | 0.0 (0) | |
| Preservative | 4.0 (9) | 5.9 (16) | 0.0 (0) | 2.6 (10) | 2.8 (5) | 0.0 (0) | | 3.7 (9) | |
| Propellant | 1. (0) | 0.0 (0) | 0.0 (0) | 0.0 (0) | 0.0 (0) | 1.0 (2) | | 0.0 (0) | |
| Raising agent | 1.8 (4) | 10.3 (28) | 2.5 (3) | 6.4 (25) | 5.5 (10) | 2.4 (5) | | 9.1 (22) | |
| Sequestrant | 23.4 (53) | 7.4 (20) | 12.7 (15) | 13.4 (52) | 38.5 (70) | 6.8 (14) | | 20.7 (50) | |
| Stabilizer | 21.6 (49) | 17.3 (47) | 15.3 (18) | 23.5 (91) | 26.9 (49) | 8.3 (17) | | 36.0 (87) | |
| Any cosmetic additive | 40.5 (92) | 45.6 (124) | 19.5 (23) | 31.7 (123) | 36.8 (67) | 16.5 (34) | | 43.8 (106) | |
| Any additive | 56.8 (129) | 61.0 (166) | 26.3 (31) | 40.7 (158) | 68.1 (124) | 24.3 (50) | | 51.7 (125) | |
| ^1^ Values presented in %(n); PDR = People’s Democratic Republic | | | | | | |  | |  |

| Supplemental Table 5. Proportion of commercially produced complementary foods containing non-permitted additives, by country^1^ | | | | | | | | | |
| --- | --- | --- | --- | --- | --- | --- | --- | --- | --- |
|  | Cambodia  (n=227) | Indonesia  (n=272) | Laos PDR  (n=118) | Malaysia  (n=388) | Philippines  (n=182) | Thailand  (n=206) | | Viet Nam  (n=242) | |
| Acidity regulator | 12.3 (28) | 12.1 (33) | 11.0 (13) | 11.9 (46) | 5.5 (10) | 11.2 (23) | | 16.5 (40) | |
| Anticaking agent | 1.8 (4) | 1.1 (3) | 0.0 (0) | 3.4 (13) | 2.8 (5) | 0.5 (1) | | 5.0 (12) | |
| Antifoaming agent | 0.0 (0) | 0.0 (0) | 0.0 (0) | 0.0 (0) | 0.0 (0) | 0.0 (0) | | 0.0 (0) | |
| Antioxidant | 7.1 (16) | 47.1 (128) | 11.9 (14) | 6.4 (25) | 5.0 (9) | 7.8 (16) | | 7.0 (17) | |
| Bleaching agent | 0.0 (0) | 0.4 (1) | 0.0 (0) | 0.0 (0) | 0.0 (0) | 0.0 (0) | | 0.0 (0) | |
| Bulking agent | 0.9 (2) | 0.4 (1) | 0.0 (0) | 0.3 (1) | 0.0 (0) | 1.9 (4) | | 0.0 (0) | |
| Carbonating agent | 0.0 (0) | 0.0 (0) | 0.0 (0) | 0.0 (0) | 0.0 (0) | 0.0 (0) | | 0.0 (0) | |
| Carrier | 1.8 (4) | 0.4 (1) | 9.3 (11) | 0.3 (1) | 0.0 (0) | 6.3 (13) | | 0.0 (0) | |
| Colour | 10.1 (23) | 3.3 (9) | 2.5 (3) | 7.2 (28) | 4.4 (8) | 3.9 (8) | | 0.8 (2) | |
| Colour retention agent | 2.6 (6) | 0.4 (1) | 0.0 (0) | 1.0 (4) | 1.7 (3) | 0.0 (0) | | 0.8 (2) | |
| Emulsifier | 17.2 (39) | 31.3 (85) | 12.7 (15) | 10.3 (40) | 12.1 (22) | 6.8 (14) | | 11.6 (28) | |
| Emulsifying salt | 7.9 (18) | 1.8 (5) | 2.5 (3) | 3.1 (12) | 2.8 (5) | 1.0 (2) | | 5.8 (14) | |
| Firming agent | 7.1 (16) | 0.0 (0) | 0.0 (0) | 1.8 (7) | 3.3 (6) | 0.5 (1) | | 4.1 (10) | |
| Flavour enhancer | 5.3 (12) | 0.0 (0) | 0.0 (0) | 1.6 (6) | 3.3 (6) | 1.5 (3) | | 5.4 (13) | |
| Flour treatment agent | 3.5 (8) | 0.7 (2) | 0.0 (0) | 1.3 (5) | 1.7 (3) | 0.5 (1) | | 5.0 (12) | |
| Foaming agent | 0.4 (1) | 0.0 (0) | 0.0 (0) | 0.3 (1) | 1.1 (2) | 1.5 (3) | | 0.0 (0) | |
| Gelling agent | 0.9 (2) | 0.4 (1) | 0.0 (0) | 0.3 (1) | 0.0 (0) | 1.9 (4) | | 0.0 (0) | |
| Glazing agent | 0.9 (2) | 0.4 (1) | 0.0 (0) | 0.3 (1) | 0.0 (0) | 1.9 (4) | | 0.0 () | |
| Humectant | 9.3 (21) | 1.5 (4) | 4.2 (5) | 5.2 (20) | 6.0 (11) | 2.9 (6) | | 6.6 (16) | |
| Packaging gas | 0.0 (0) | 0.0 (0) | 0.0 (0) | 0.0 (0) | 0.0 (0) | 1.0 (2) | | 0.0 (0) | |
| Preservative | 0.4 (1) | 5.5 (15) | 0.0 (0) | 1.8 (7) | 2.2 (4) | 0.0 (0) | | 2.9 (7) | |
| Propellant | 0.0 (0) | 0.0 (0) | 0.0 (0) | 0.0 (0) | 0.0 (0) | 1.0 (2) | | 0.0 (0) | |
| Raising agent | 0.9 (2) | 9.9 (27) | 2.5 (3) | 5.4 (21) | 1.7 (3) | 2.4 (5) | | 7.0 (17) | |
| Sequestrant | 5.3 (12) | 1.8 (5) | 5.1 (6) | 4.6 (18) | 4.4 (8) | 1.0 (2) | | 6.6 (16) | |
| Stabilizer | 11.9 (27) | 4.0 (11) | 2.5 (3) | 6.7 (26) | 8.8 (16) | 2.4 (5) | | 8.3 (20) | |
| Sweetener | 0.0 (0) | 0.0 (0) | 0.0 (0) | 0.0 (0) | 0.0 (0) | 1.0 (2) | | 0.0 (0) | |
| Thickener | 14.5 (33) | 16.5 (45) | 5.1 (6) | 10.1 (39) | 18.1 (33) | 6.3 (13) | | 11.2 (27) | |
| Any non-permitted additive | 36.6 (83) | 53.7 (146) | 22.9 (27) | 23.5 (91) | 26.9 (49) | 18.5 (38) | | 24.8 (60) | |
| ^1^ Values presented in %(n); PDR = People’s Democratic Republic | | | | | | |  | |  |


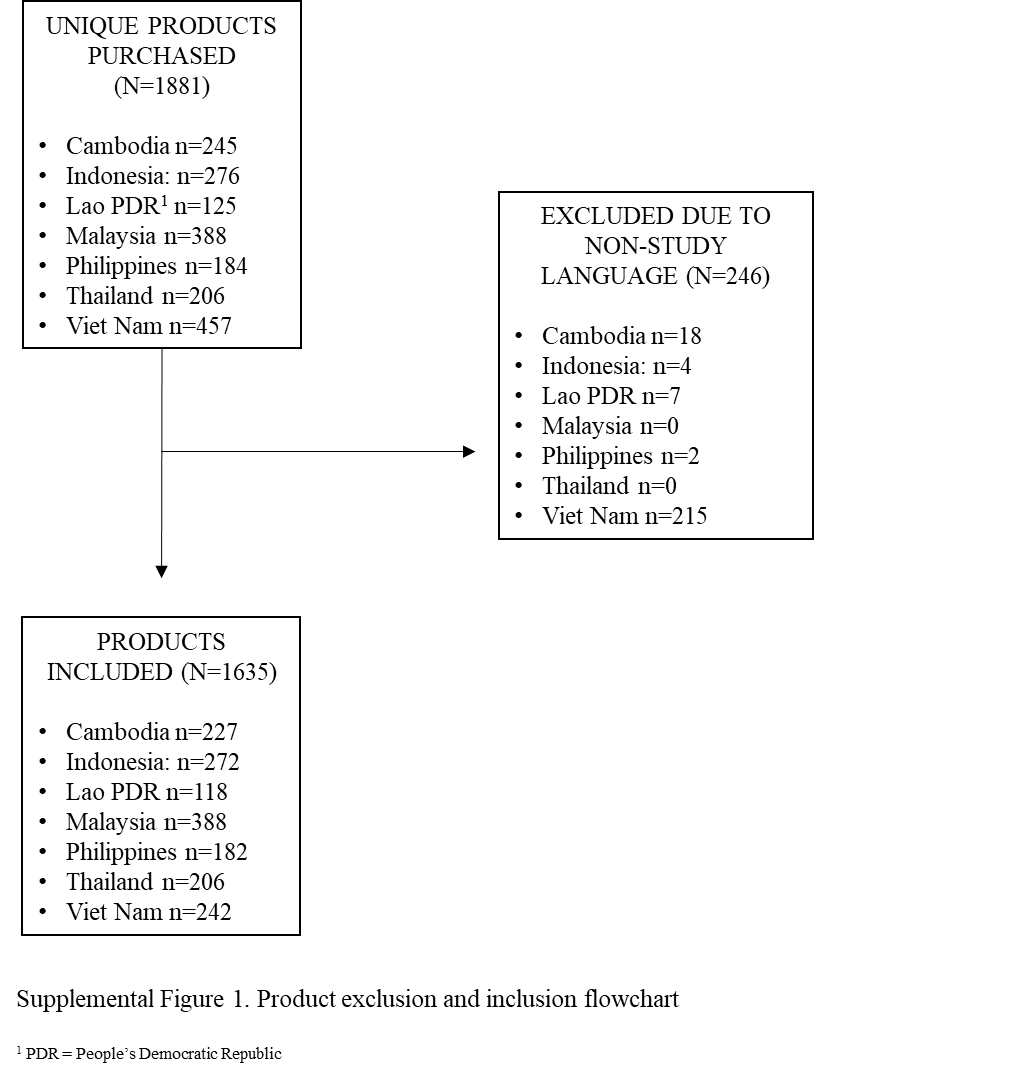

Supplement: multimedi component 1 [file mmc1.docx]
